# Discovery of a small protein-encoding *cis*-regulatory overlapping gene of the tumor suppressor gene *Scribble* in humans

**DOI:** 10.1038/s42003-021-02619-8

**Published:** 2021-09-17

**Authors:** Yuhta Nomura, Naoshi Dohmae

**Affiliations:** Biomolecular Characterization Unit, RIKEN Center for Sustainable Resource Science, 2-1 Hirosawa, Wako, Saitama, 351-0198 Japan

**Keywords:** Proteomics, Proteome informatics, Cancer genomics, Open reading frames

## Abstract

Intensive gene annotation has revealed many functional and regulatory elements in the human genome. Although eukaryotic protein-coding genes are generally transcribed into monocistronic mRNAs, recent studies have discovered additional short open reading frames (sORFs) in mRNAs. Here, we performed proteogenomic data mining for hidden proteins categorized into sORF-encoded polypeptides (SEPs) in human cancers. We identified a new SEP-encoding overlapping sORF (oORF) on the cell polarity determinant *Scribble* (*SCRIB*) that is considered a proto-oncogene with tumor suppressor function in Hippo-YAP/TAZ, MAPK/ERK, and PI3K/Akt/mTOR signaling. Reanalysis of clinical human proteomic data revealed translational dysregulation of both *SCRIB* and its oORF, *oSCRIB*, during carcinogenesis. Biochemical analyses suggested that the translatable *oSCRIB* constitutively limits the capacity of eukaryotic ribosomes to translate the downstream *SCRIB*. These findings provide a new example of *cis*-regulatory oORFs that function as a ribosomal roadblock and potentially serve as a fail-safe mechanism to normal cells for non-excessive downstream gene expression, which is hijacked in cancer.

## Abstract (web summary)

Yuhta Nomura and Naoshi Dohmae report the discovery of a small protein-coding gene that overlaps the tumor suppressor gene *Scribble*. Their data suggest that the overlapping gene, *oSCRIB*, limits the translation of downstream *Scribble* and may have important implications in cancer.

## Introduction

More accurate and detailed annotation of the human genome has shed light on many functional and regulatory elements, including protein-coding genes and noncoding RNA genes (e.g., ribosomal RNA, transfer RNA, and microRNA genes). Eukaryotic protein-coding genes are generally transcribed into monocistronic messenger RNAs (mRNAs), each with a single protein-coding open-reading frame (ORF) and two untranslated regions (UTRs: 5′-UTR and 3′-UTR) for their subsequent translation^1^. Contrary to this view, there is growing evidence that eukaryotic mRNAs can code for more than one protein, indicating the existence of exceptions to the dogma^2–5^. In fact, recent studies have uncovered many classes of additional ORFs (viz., alternative ORFs, altORFs), such as short/small ORFs (sORFs/smORFs), in mRNAs^6–10^, which are translated on ribosomes and may produce small proteins or polypeptides <150 amino acids, namely sORF-encoded polypeptides (SEPs)^6^. Based on their relative position against the primary ORF (viz., reference protein-coding sequence, refCDS), which is usually the longest ORF per transcript, they are currently categorized into three major groups: upstream ORFs (uORFs or altORFs^5′-UTR^), overlapping ORFs (oORFs or altORFs^CDS^), and downstream ORFs (dORFs or altORFs^3^^′^^-UTR^)^3^. Interestingly, it has been reported that approximately 50% of mRNAs potentially code for at least one uORF or oORF, and the majority of oORF-containing mRNAs have also at least one potential uORF in humans and mice^11,12^. In addition, there is emerging evidence that uORFs and oORFs, both of which are altORFs, act as translational *cis*-regulatory elements of refCDSs^12,13^ and are closely associated with tumor-initiating and tumor-progressing unconventional translation in cancer^13,14^. These studies on altORFs are opening new avenues for therapeutic interventions in cancer treatment. Furthermore, the presence of altORFs in some long noncoding RNAs and circular RNAs has also been reported^6,8,15–17^, termed altORFs^lnc^ and altORFs^circ^, respectively. These striking examples indicate a higher density of genetic information in the human genome and motivate our research on SEP gene discovery.

Mass spectrometry (MS)-based proteomics and ribosome profiling (or Ribo-Seq)-based translatomics are both becoming powerful tools for novel protein/peptide discovery^6–10^. In particular, the former approach has the capability to conclusively identify peptides and proteins by their direct detection^18^. This has permitted the simultaneous identification of human peptides and proteins encoded by 17,294 genes, which account for approximately 84% of protein-coding genes previously annotated in the human genome^15^. On the other hand, there is inherent dependence of current MS-based proteomics on predefined databases of protein sequences and/or translated nucleotide sequences for protein identification. Therefore, a combination of high-quality data on both proteomic mass spectra and genomic/transcriptomic nucleotide sequences (or ORFeomes) is indispensable for the more comprehensive identification of human proteomes^19–21^. This proteogenomic approach still promises to uncover previously ignored dark proteomes and peptidomes, including novel SEPs.

In this study, we performed proteogenomic data mining for hidden SEP-encoding genes in the human genome through the use of publicly available high-quality datasets of human cancer cell lines, which were obtained from MS-based proteomics and transcriptomic RNA sequencing (RNA-Seq). Our study successfully demonstrated the existence of a new SEP-encoding oORF on the cell polarity-determining scaffold protein gene *Scribble* (*SCRIB*), which is considered a proto-oncogene with tumor suppressor function in antitumorigenic Hippo-YAP/TAZ, Ras/Raf/MEK/ERK (MAPK/ERK), and PI3K/Akt/mTOR and proapoptotic c-Myc-induced signaling pathways^22–29^. Together with the results from our clinical human proteomic data reanalysis and biochemical analysis, we report that the translatable oORF on *SCRIB*, *oSCRIB*, is a *cis*-regulatory oORF potentially providing a fail-safe mechanism to normal cells for nonexcessive downstream *SCRIB* expression, whereas the mechanism was dysregulated in cancer cells for their survival and proliferation.

## Results

### Transcriptomic data mining for hidden protein-coding potential of the human genome

**Fig. 1:**
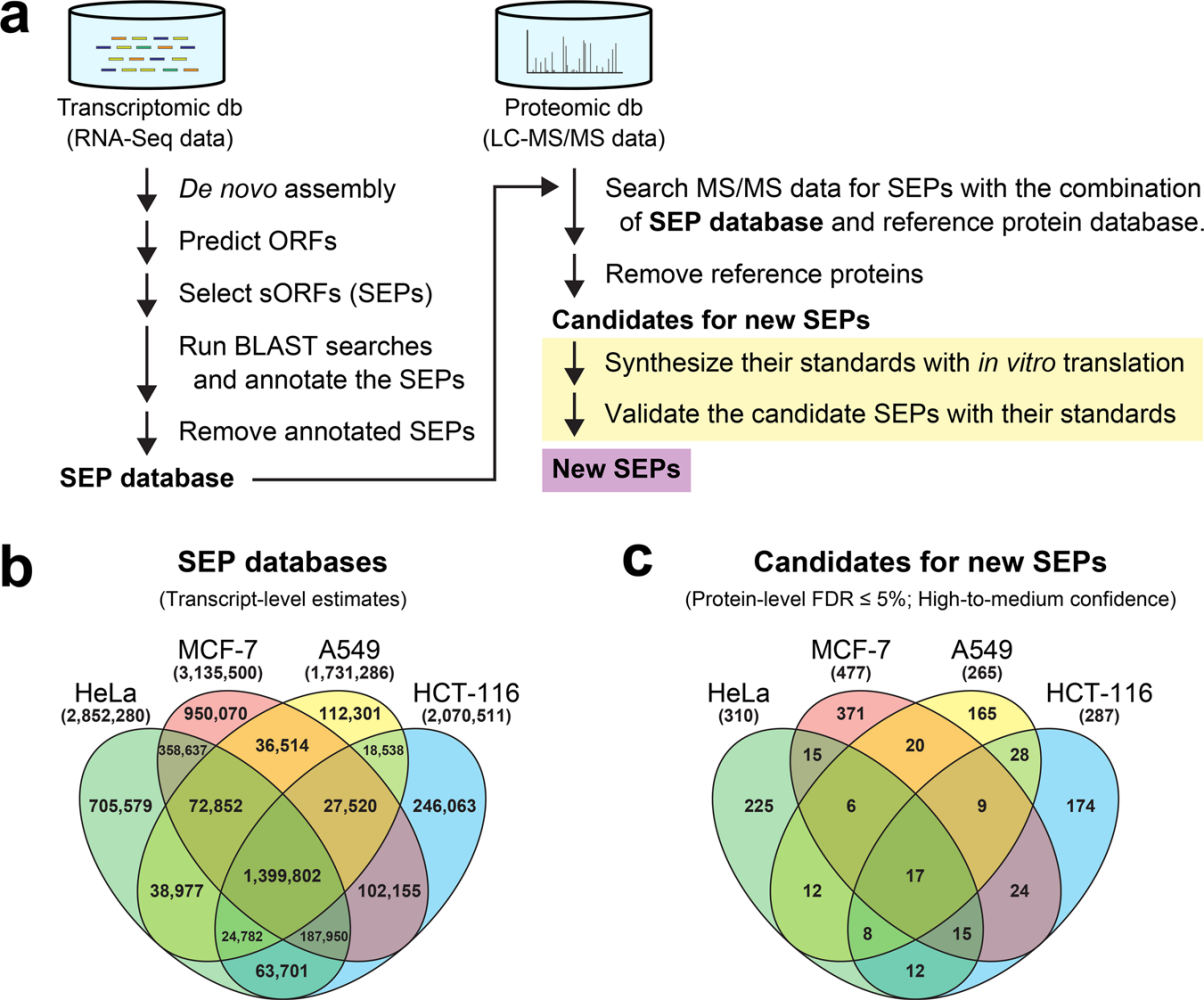
Proteogenomic data mining for new SEP proteins in human cell lines. **a** Proteogenomics workflow for SEP gene discovery in this study. This workflow used a cell-free translation system for authentic protein/peptide production as the final step (yellow highlight) providing conclusive evidence of mass spectrometric protein-identification results, which are normally obtained by probability-based selection in automated search engines (e.g., Mascot). **b, c** Venn diagrams showing the number of SEP candidates detected in human cell lines at the transcriptome (**b**) and proteome (**c**) levels.

We performed proteogenomic data mining in human cancer cell lines, as illustrated in Fig. 1a. High-quality transcriptomic data that can yield high sequence coverage can achieve a more comprehensive estimation of proteomes. In addition, a combination of proteomic and transcriptomic data from the same cell line would maximize the predictive power of proteogenomics (i.e., proteotranscriptomics)-based gene discovery. Therefore, with several publicly available RNA-Seq datasets of the human transcriptome from cancer cell lines (HeLa, MCF-7, A549, and HCT-116; three biological replicates for each cell line), we first performed de novo transcriptome assemblies and searched the resulting contigs for possible ORFs. Additionally, we also extracted potentially translatable ORFs from a nonredundant database of human reference transcript sequences (RefSeq Transcripts^30^; RefSeq assembly accession GCF_000001405.39), which was prepared from the Genome Reference Consortium Human Build 38 patch release 13 (GRCh38.p13)^31^ by the National Center for Biotechnology Information (NCBI). Next, we extracted all possible sORFs possibly encoding SEPs of 10–149 amino acids that start and stop with AUG and stop codons, respectively, from the newly constructed datasets of ORFs found in cancer cell lines (HeLa, MCF-7, A549, and HCT-116) and RefSeq Transcripts. The resulting datasets of sORFs from three replicates of each cell line were aggregated into a nonredundant dataset for each cell line. The translated nucleotide sequences of sORFs, i.e., the amino acid sequences of putative SEPs, were further searched using the Basic Local Alignment Search Tool (BLAST)^32^ against a nonredundant database of human reference protein sequences (RefSeq Proteins^30^; RefSeq assembly accession GCF_000001405.39), which was also prepared from GRCh38.p13 by NCBI. These preliminary transcriptomic data analyses predicted 3,216,009, 3,704,082, 1,533,942, 2,051,239, and 1,781,513 SEP candidates in HeLa, MCF-7, A549, HCT-116, and RefSeq Transcripts, respectively. These results indicated the presence of a high degree of protein-coding potential of the human genome. Although the retrieved sequences may still include partial protein sequences, such as *N*-terminally truncated protein sequences, due to incomplete transcriptome assembly, the results indicated that almost two-thirds of these sequences, 2,204,181, 2,506,331, 1,029,024, 1,375,127, and 1,146,023 sequences in HeLa, MCF-7, A549, HCT-116, and RefSeq Transcripts, respectively, were not assigned as genes currently annotated in human RefSeq Proteins and were expected to contain real, previously unidentified SEPs. Therefore, we only retained the unassigned sequences in the SEP datasets. To improve the predictive power of proteogenomics-based gene discovery, we further integrated the SEP dataset of RefSeq Transcripts into that of each cell line and used the integrated nonredundant datasets as the custom SEP sequence databases (for HeLa, MCF-7, A549, and HCT-116) to perform further proteogenomic analyses. These custom SEP sequence databases for each line consisted of 2,852,280, 3,135,500, 1,731,286, and 2,070,511 sequences in HeLa, MCF-7, A549, and HCT-116, respectively (Fig. 1b). Furthermore, we also prepared the complete dataset for which we integrated the SEP datasets of all cell lines (HeLa, MCF-7, A549, and HCT-116) and RefSeq Transcripts into a single nonredundant database; it ultimately consisted of 4,345,441 sequences.

### Proteomic data mining for new SEPs in humans

High-quality MS-based proteomic data that comprise human proteins with high coverage are also indispensable for a more comprehensive identification of human proteomes. Recently optimized shotgun proteomics with off-line high-pH reversed-phase fractionation of peptides and subsequent multiple injections of each fraction into online low-pH liquid chromatography-tandem MS (LC–MS/MS) accomplished the generation of in-depth human proteomes^33,34^. The secondary use of these high-quality datasets is also important for mining dark proteomes and peptidomes. Therefore, we also obtained the raw datasets comprising tryptic mass spectra of the human proteome from cancer cell lines (HeLa, MCF-7, A549, and HCT-116; two biological replicates for each cell line) deposited in ProteomeXchange^35^ (dataset identifier PXD004452) by Bekker-Jensen et al.^34^ for further proteogenomic analyses.

The raw tryptic mass spectra files were first processed to recalibrate precursor masses and convert the files into Mascot Generic Format (MGF) files, which contain MS/MS peak lists and experimental parameters, with Thermo Proteome Discoverer (Thermo Fisher Scientific). The MGF files were then submitted to an in-house Mascot Server^36^ through Thermo Proteome Discoverer and searched against the two different sequence databases in parallel, i.e., the combination of human RefSeq Proteins and the custom SEP sequence database (for HeLa, MCF-7, A549, or HCT-116), with target decoy-based false-discovery rate (FDR) filtering^37,38^ and parameters described in the “Methods” section. The peptide-spectrum matching (PSM) by the Mascot algorithm predicted the inclusion of 258,123, 166,787, 175,353, and 200,071 peptide sequences in the HeLa, MCF-7, A549, and HCT-116 proteomes, respectively, each with an FDR of ≤1% at the peptide level. Among the detected peptides, approximately 0.5–0.7% of peptides (HeLa, 1738; MCF-7, 1,166; A549, 889; and HCT-116, 983) were assigned to SEP candidates (HeLa, 1709; MCF-7, 1120; A549, 861; and HCT-116, 943) derived from the custom SEP sequence databases, whereas the remaining peptides (HeLa, 256,385; MCF-7, 165,621; A549, 174,464; and HCT-116, 199,088) were assigned to known proteins (HeLa, 11,186; MCF-7, 10,939; A549, 11,195; and HCT-116, 11,624) listed in human RefSeq Proteins. On the other hand, when using the single complete database into which we integrated the SEP datasets of all cell lines (HeLa, MCF-7, A549, and HCT-116) and RefSeq Transcripts, Mascot algorithm-assisted PSM predicted the slightly smaller number of peptides (256,228) in the HeLa proteome, among which 2225 and 254,003 peptides were assigned to 2178 SEP candidates and 11,130 known proteins, respectively, each with an FDR of ≤1% at the peptide level. These results indicated the limited advantage of the single complete database as compared with cell-type-matched databases in the detection power of proteomes. Since our SEP discovery was conducted within a commercial proteome informatics pipeline (i.e., Thermo Proteome Discoverer, as described above), a class-specific FDR estimation recommended by Nesvizhskii^20^ was not taken into consideration. Our results were instead filtered to maintain a low FDR of ≤1% at the peptide level, thereby ensuring high confidence in the detected peptides. In addition, approximately 96–97% and 42–47% of the detected peptides derived from human RefSeq Proteins and the custom SEP sequence database, respectively, had lower FDRs of ≤0.5%.

**Fig. 2:**
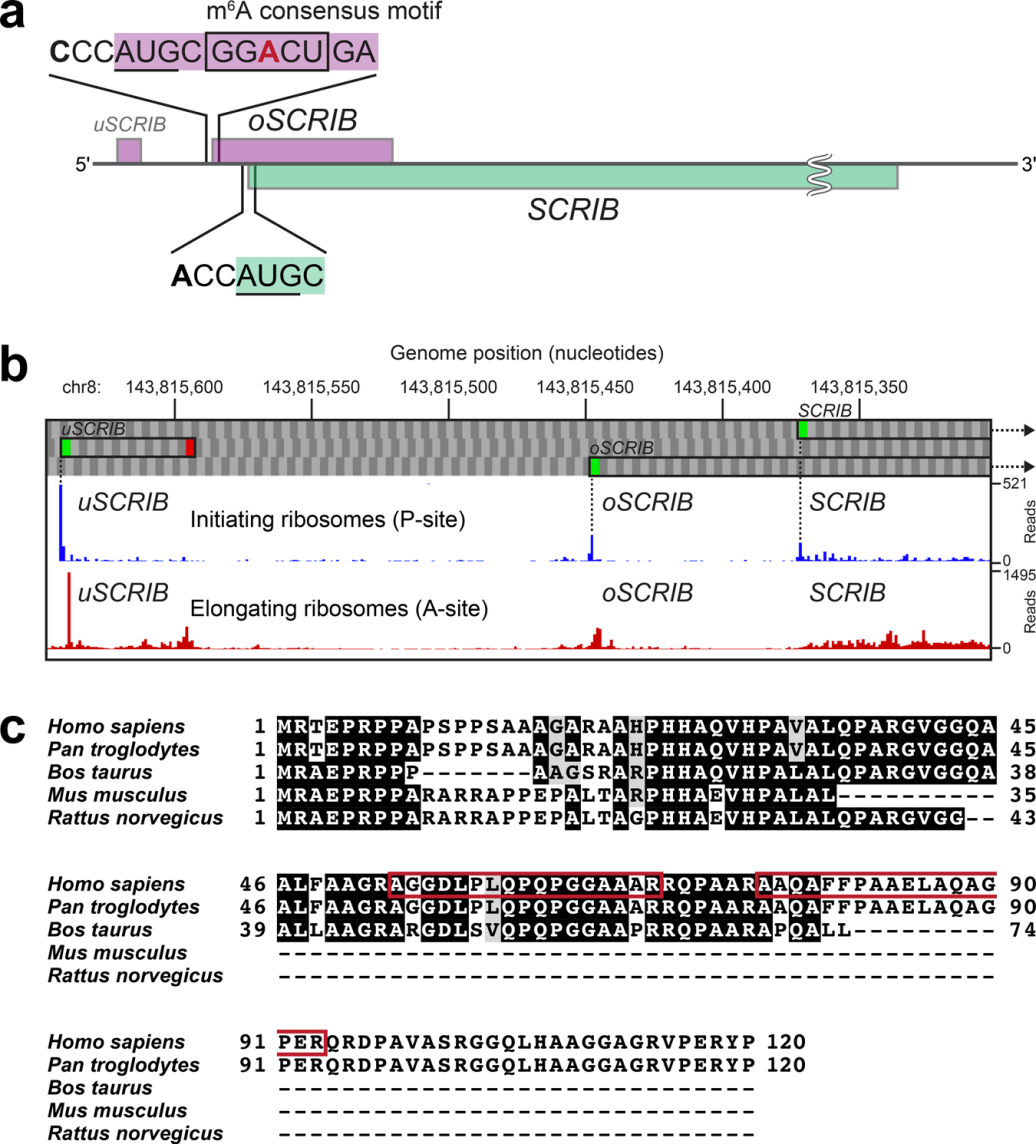
Investigation of the newly discovered oSCRIB protein. **a** The transcript encoding the main SCRIB protein contains additional unidentified sORFs (*uSCRIB* and *oSCRIB*). The oSCRIB-coding region overlapped the downstream out-of-frame *SCRIB* gene in the human genome. Translational start codons (AUGs) of *oSCRIB* and *SCRIB* and the surrounding sequences in humans are shown. The most prevalent sequence (GGACU) for the reversible epitranscriptomic m^6^A modification and the resultant m^6^A-dependent start codon selection^42,43,58,73^ are shown in the box. **b** Survey of publicly available Ribo-Seq data. The aggregated profiles of initiating and elongating ribosomes were obtained from GWIPS-viz^74^ (https://gwips.ucc.ie). **c** Translation products of *oSCRIB* in humans (*Homo sapiens*), chimpanzees (Pan troglodytes), cattle (*Bos taurus*), mice (*Mus musculus*), and rats (*Rattus norvegicus*). The protein sequences were aligned and colored using the GenomeNet ClustalW 2.1 and EMBnet BoxShade 3.21 servers (https://www.genome.jp/tools-bin/clustalw and https://embnet.vital-it.ch/software/BOX_form.html). Red boxes indicate the amino acid sequences of tryptic peptides detected in LC–MS/MS analyses as shown in Figs. 3 and 4.

We next evaluated the results at the protein level. Among the detected SEP candidates (HeLa, 1709; MCF-7, 1120; A549, 861; and HCT-116, 943) described above, 35, 82, 37, and 56 candidates in the HeLa, MCF-7, A549, and HCT-116, respectively, had a relatively low FDR of ≤1% (high confidence) at the protein level; 275, 395, 228, and 231 candidates in the HeLa, MCF-7, A549, and HCT-116, respectively, had a relaxed FDR of >1% but still maintained an FDR of ≤5% (medium confidence); the remaining candidates (HeLa, 1399; MCF-7, 643; A549, 596; and HCT-116, 656) each had a high FDR of >5% (low confidence). The high-to-medium-confidence candidates (HeLa, 310; MCF-7, 477; A549, 265; and HCT-116, 287) included 17 SEP candidates (7 high- and 10 medium-confidence candidates) that were detected in all the human cell lines described above (Fig. 1c). Interestingly, the 7 high-confidence candidates included two SEP candidates with a protein-level FDR of 0%, both of which were detected not only in the HeLa proteome but also in the other human cell lines mentioned above. This suggests the presence of the two SEPs in human cells. One of the two SEP-encoding sORFs overlapped with the cell-polarity protein gene *Scribble* (*SCRIB*)^28^ in the human genome; this overlapping sORF had not previously been annotated. The spectrum-centric PSM approach described above predicted the existence of two peptides unique to this SEP, AGGDLPLQPQPGGAAAR and AAQAFFPAAELAQAGPER, with very low peptide-level FDRs of 0.0018% and 0%, respectively. Furthermore, an alternative peptide-centric approach by PepQuery also retrieved the same unique peptides (PepQuery *P*-value, 0.001%) from the publicly available proteomic data (MGF format) under a P-value cutoff of 1%, where well-controlled FDRs are produced^39^. We thus designated this SEP-encoding sORF (120 amino acids in length) as an oORF on *SCRIB* or *oSCRIB* (Fig. 2a and Supplementary Table 1). On the other hand, the existence of the other SEP (63 amino acids in length) was also supported by the detection of NDDIPEQDSLGLSNLQK by the Mascot algorithm with very low peptide-level FDR of 0%. The PepQuery also retrieved the same unique peptide from the MGF files (PepQuery *P*-value, 0.001%; *P*-value cutoff, 1%). However, this SEP was equivalent to an evolutionarily conserved uORF of the *McKusich–Kaufman Syndrome* (*MKKS*) gene, which was previously reported as *uMKKS1*^40^ (Supplementary Table 1) and whose translation product was also detected by MS-based proteomics^41^, although it has not been included in human RefSeq Proteins. Accordingly, we focused on the newly discovered oSCRIB protein in this study.

### Further investigation of the newly discovered oSCRIB protein

It is worth noting that approximately 80% of the oSCRIB-coding region (287 of 363 bp) overlapped with a downstream out-of-frame *SCRIB* gene (two transcript variants, 1 and 2, 4968 bp and 4893 bp, respectively) in the human genome (Fig. 2a). These two adjacent genes, *oSCRIB* and *SCRIB*, correspond to oORF (or altORF^CDS^) and refCDS, respectively; they share the same genomic region and are transcribed into a single bicistronic mRNA, after which they are translated into proteins with different translational start codons. A survey of the *oSCRIB* sequence itself and its upstream region in the human genome further indicated the existence of an internal epitranscriptomic *N*^6^-methyladenosine (m^6^A) consensus motif (GGACU)^42,43^ and an unknown, potentially translatable uORF of *SCRIB* (*uSCRIB*), respectively (Fig. 2a). Since our custom SEP sequence databases included the translated *uSCRIB* sequence, we checked the publicly available MS-based proteomic data again for uSCRIB. Although both oSCRIB and SCRIB were successfully detected in these proteomes with protein-level FDRs of 0%, the translation product of *uSCRIB* (MRSRRRRRSPRFLRV, 15 amino acids in length) was not detected. This was likely due to its low abundance and/or tryptic digestion of its arginine-rich region during sample preparation. In support of our proteogenomic data mining, the publicly available Ribo-Seq-based translatomic data also implied the translational initiation and elongation of ribosomes at *uSCRIB*, *oSCRIB*, and *SCRIB* in human cancer cell lines (Fig. 2b). Additionally, other mammalian genomes were found to potentially conserve oSCRIB homologous proteins (Fig. 2c).

### Conclusive validation of the identification of oSCRIB protein

A Mascot server performs the probability-based protein identification in silico by fitting experimental MS/MS data into theoretical MS/MS models that are constructed by user-selected protein-sequence datasets^36^. Therefore, it is necessary to validate the identification of SEPs with their authentic proteins. The newly discovered oSCRIB was the target of this study, but the previously reported uMKKS1 was used as a positive control in subsequent investigations.

**Fig. 3:**
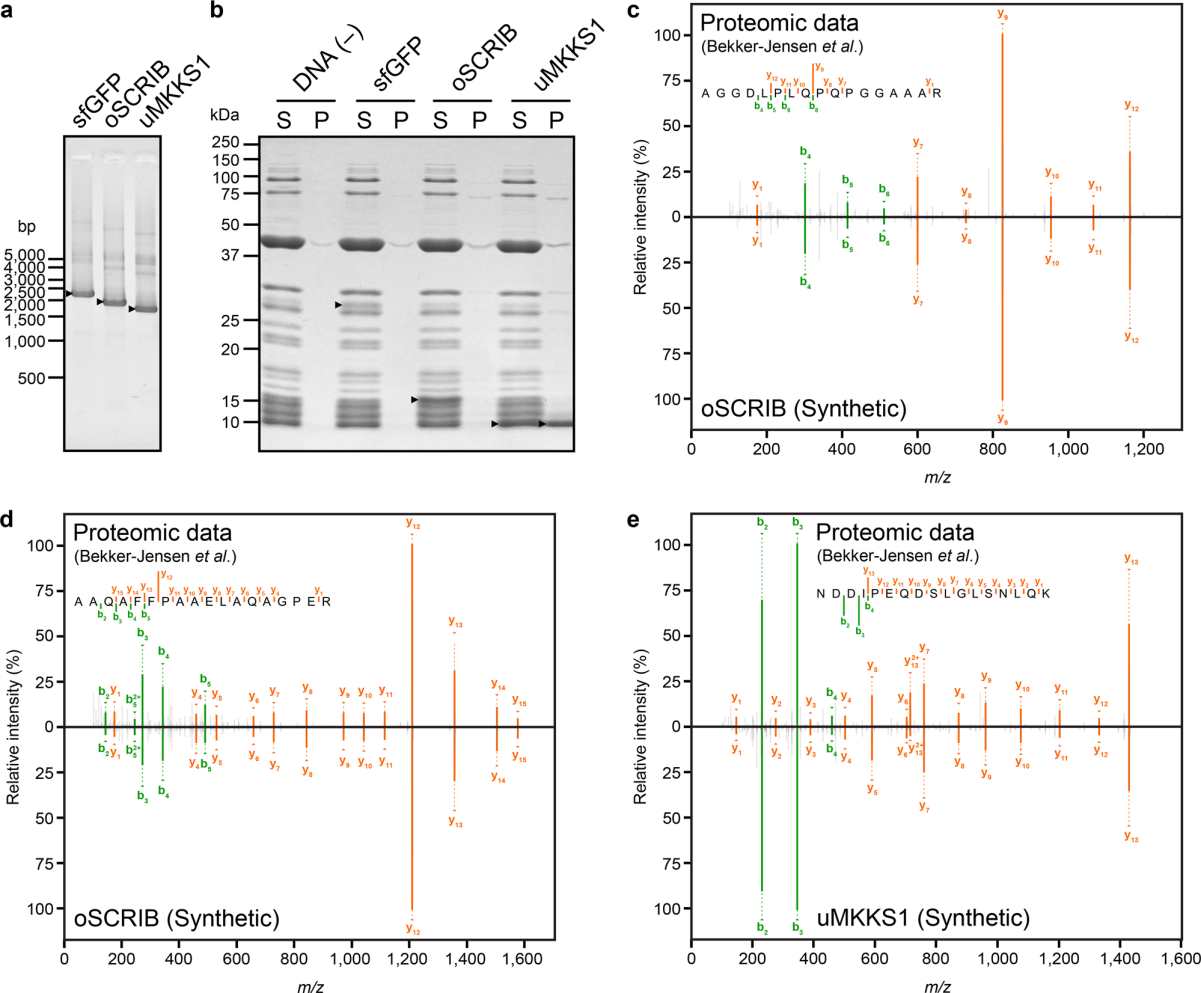
Cell-free translation system-assisted rapid validation of new SEP protein identification. **a** Linear DNA fragments (*sfGFP*, *oSCRIB*, and *uMKKS1*) prepared for in vitro production of authentic proteins/peptides were analyzed by agarose gel electrophoresis. The DNA fragments were visualized under ultraviolet light on gels stained with GelRed, and the positions of molecular standards are indicated. *Arrowheads* indicate the bands of the DNA fragments. **b** SDS-PAGE analysis of in vitro-synthesized proteins (sfGFP, oSCRIB, and uMKKS1). Coupled in vitro reactions of transcription and translation with and without the DNA fragment encoding sfGFP protein were used as positive and negative controls of the reactions, respectively. The gel was stained with Coomassie Brilliant Blue G-250, and the positions of molecular standards are indicated. *Arrowheads* indicate the bands of proteins synthesized in vitro, whereas other bands were derived from enzymes for transcription and translation. The following abbreviations are used: S, soluble fractions; and P, insoluble fractions. **c–e** Mirror plots of MS/MS spectra obtained by LC–MS/MS in DDA mode. The spectra included in publicly available proteomic data on HeLa cells deposited in the ProteomeXchange (dataset identifier PXD004452) by Bekker-Jensen et al.^34^ are shown above, whereas those of the corresponding authentic peptides obtained by tryptic digestion of in vitro-synthesized proteins are inverted and shown below. The spectra included the profiles of the product ions generated by HCD of divalent precursor ions [M + 2H]^2+^ that were derived from tryptic peptides: AGGDLPLQPQPGGAAAR (**c**) and AAQAFFPAAELAQAGPER (**d**) of oSCRIB protein; and NDDIPEQDSLGLSNLQK (**e**) of uMKKS1 protein.

For the simultaneous synthesis of multiple authentic proteins in small quantities, we employed a commercially available in vitro translation system based on bacterial 70 S ribosomes, called Protein synthesis Using Recombinant Elements (PURE) system^44^. We first constructed linear DNA fragments that were used as templates for in vitro transcription and translation (Fig. 3a). The DNA fragments contained a bacteriophage T7 RNA polymerase promoter, bacterial ribosome-binding site called a Shine–Dalgarno sequence, and either the sORF encoding oSCRIB or uMKKS1 or the ORF encoding a superfolder green fluorescent protein (sfGFP)^45^ as a positive control of in vitro translation. Then in vitro translation coupled with transcription was performed with the PURE system in the presence of the DNA fragments, and the synthesized proteins were detected by sodium dodecyl sulfate–polyacrylamide gel electrophoresis (SDS-PAGE). The results showed their successful expression in vitro; oSCRIB and sfGFP proteins were only detected in the soluble fraction, whereas uMKKS1 protein was found in both the soluble and insoluble fractions (Fig. 3b).

Next, we performed in-gel tryptic digestion of the in vitro-synthesized oSCRIB and uMKKS1 proteins excised from the SDS-PAGE gels. The resultant peptides were analyzed as authentic standards by LC–MS/MS under data-dependent acquisition (DDA) control^46^. These MS/MS spectra included the profiles of the product ions generated by higher-energy collisional dissociation (HCD)^46,47^ of divalent precursor ions [M + 2H]^2+^ that were derived from tryptic peptides as follows: AGGDLPLQPQPGGAAAR (*m/z* 788.41425) and AAQAFFPAAELAQAGPER (*m/z* 922.96613) of the authentic oSCRIB protein (Fig. 3c, d), and NDDIPEQDSLGLSNLQK (*m/z* 943.45764) of the authentic uMKKS1 protein (Fig. 3e). Manual comparison of these MS/MS spectra with those of the corresponding peptides obtained from the above-mentioned proteome dataset (PXD004452) confirmed their equivalent fragmentation patterns (Fig. 3c–e). Therefore, this inspection provided evidence for the existence of oSCRIB and uMKKS1 proteins in human cell lines.

**Fig. 4:**
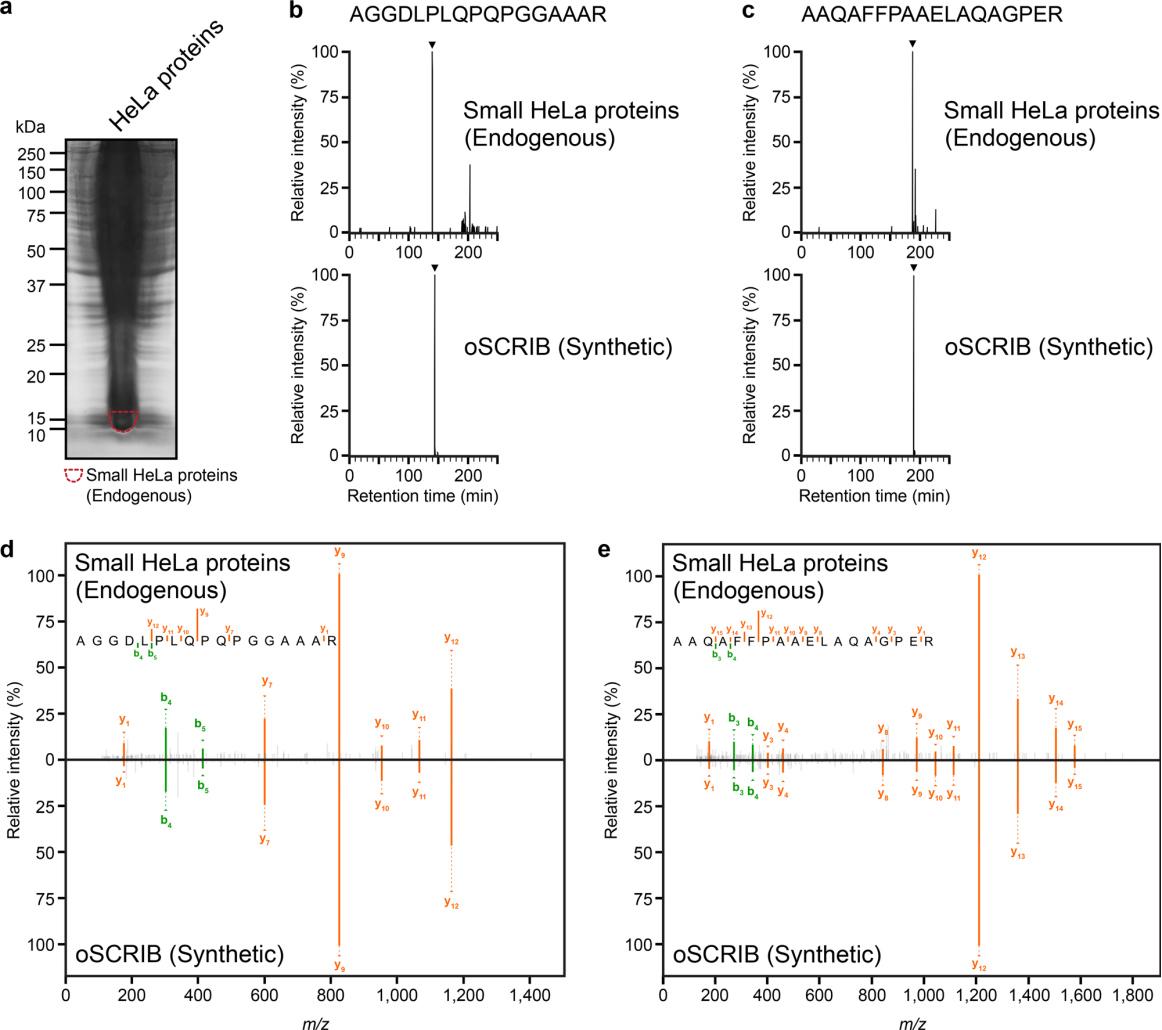
Further validation of the newly identified oSCRIB protein. **a** SDS-PAGE profile of endogenous HeLa proteins. The gel was stained with Coomassie Brilliant Blue G-250, and the positions of molecular standards are indicated. The broken circle indicates the protein fraction used for LC–MS/MS analysis, where small HeLa proteins of approximately 10–15 kDa, including oSCRIB protein (12 kDa), were enriched. **b–e** The results of LC–MS/MS analysis in PRM mode. The PRM chromatograms (**b, c**) and MS/MS spectra (**d, e**) obtained from the protein fraction in (**a**) are shown above, whereas those of the corresponding authentic peptides obtained by tryptic digestion of in vitro-synthesized oSCRIB protein are shown below. The lower parts of (**d, e**) are inverted to make mirror plots. The PRM chromatograms of (**b**) show the extracted peaks containing the product ions of *m/z* 824.4100–824.4700 among the precursor ions of *m/z* 788.4155 for AGGDLPLQPQPGGAAAR and those of (**c**) show the extracted peaks containing the product ions of *m/z* 1209.6000–1209.6600 among the precursor ions of *m/z* 922.9681 for AAQAFFPAAELAQAGPER. The MS/MS spectra (**d, e**) corresponding to the peaks indicated in (**b–c**) by *arrowheads* included the profiles of the product ions generated by HCD of divalent precursor ions [M + 2H]^2+^ that were derived from tryptic peptides unique to oSCRIB protein, AGGDLPLQPQPGGAAAR (**d**) and AAQAFFPAAELAQAGPER (**e**).

Finally, to further validate the newly identified oSCRIB protein, we also extracted HeLa proteins. The extracted HeLa proteins were subjected to SDS-PAGE for protein separation (Fig. 4a), and the enrichment of small proteins approximately 10–15 kDa, which included oSCRIB protein (12 kDa), was carried out by excising the corresponding protein bands from SDS-PAGE gels. These small proteins were then digested in-gel with trypsin and the resultant peptides were analyzed by LC–MS/MS. To obtain high sensitivity and selectivity toward the detection of tryptic peptides of oSCRIB protein, we applied targeted MS/MS acquisition called parallel reaction monitoring (PRM)^48^. Our results showed the successful detection of two peptides unique to oSCRIB protein, AGGDLPLQPQPGGAAAR and AAQAFFPAAELAQAGPER, and their retention times and fragmentation patterns were identical to those of the corresponding peptides obtained from the authentic oSCRIB protein (Fig. 4b–e). Therefore, our results confirmed the existence of oSCRIB protein in human cells at the protein level.

### The newly discovered *oSCRIB* is a translational inhibitory element of *SCRIB*

Recent studies have revealed that the downstream *SCRIB* is a double-agent gene as a proto-oncogene with tumor suppressor function (POTSF) in normal cells^26,27,29^. In fact, *SCRIB* is reported to be overexpressed at the mRNA and protein levels in multiple human cancers, including uterine and breast cancers, and the resultant mislocalization of SCRIB protein promotes tumorigenesis^23,26^.

**Fig. 5:**
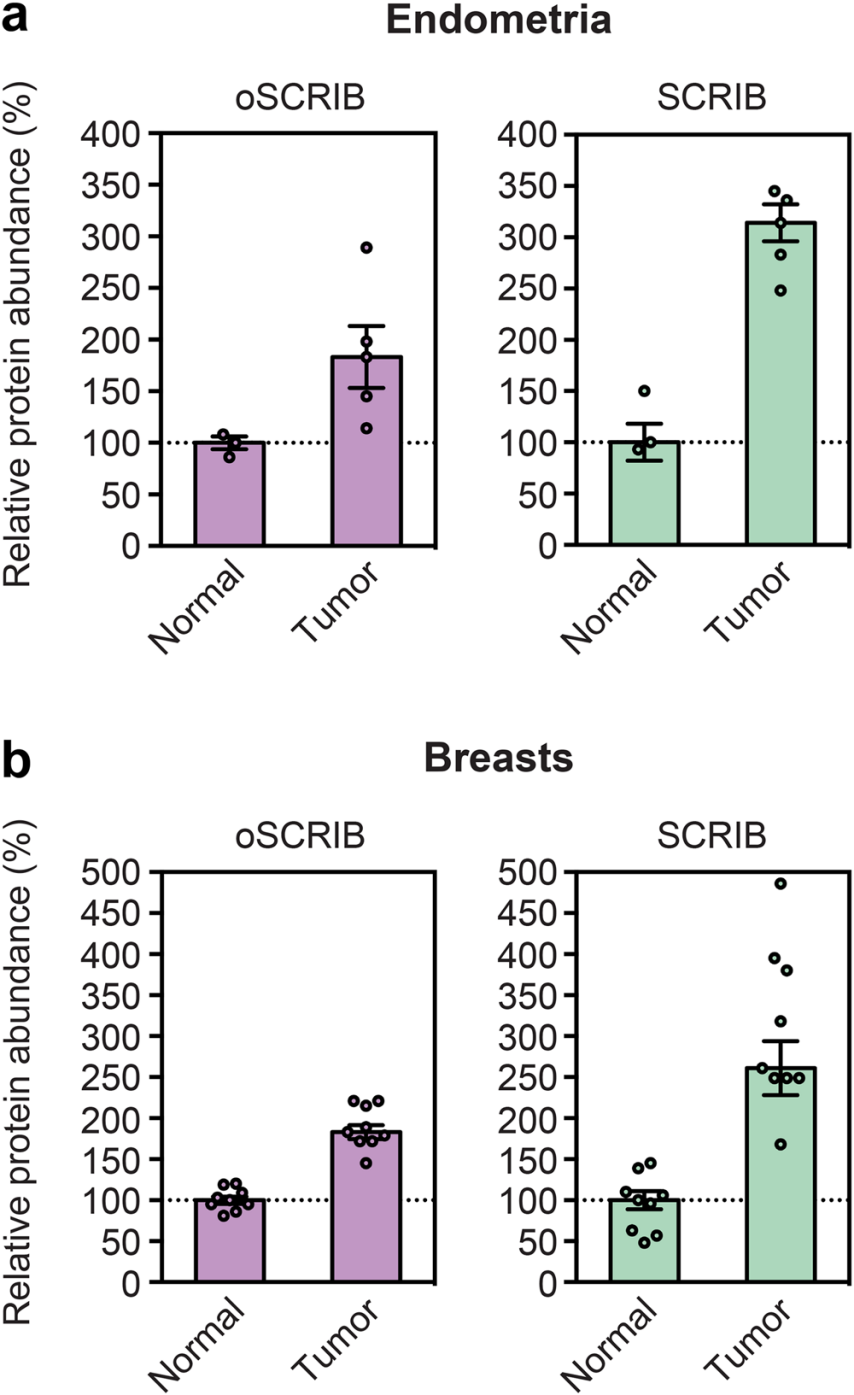
The relationship between *oSCRIB* and *SCRIB* expression in carcinogenesis. Reanalysis of publicly available quantitative proteomic data on human primary tumor and adjacent normal tissues from endometria (**a**) and breasts (**b**). The data calculated by Thermo Proteome Discoverer (Thermo Fisher Scientific) are the median ± SE of independent samples.

To investigate the relationship between *oSCRIB* and *SCRIB* expression in carcinogenesis, we reanalyzed publicly available tandem mass tag (TMT) isobaric labeling-based quantitative proteomic data on human primary tumor and adjacent normal tissues from endometria^49^ and breasts^50^. The expression levels of oSCRIB and SCRIB proteins were simultaneously increased in human endometrial (Fig. 5a) and breast (Fig. 5b) primary tumors as compared with adjacent normal tissues. This suggests translational dysregulation of both *oSCRIB* and *SCRIB* on *oSCRIB*–*SCRIB* mRNAs during carcinogenesis.

**Fig. 6:**
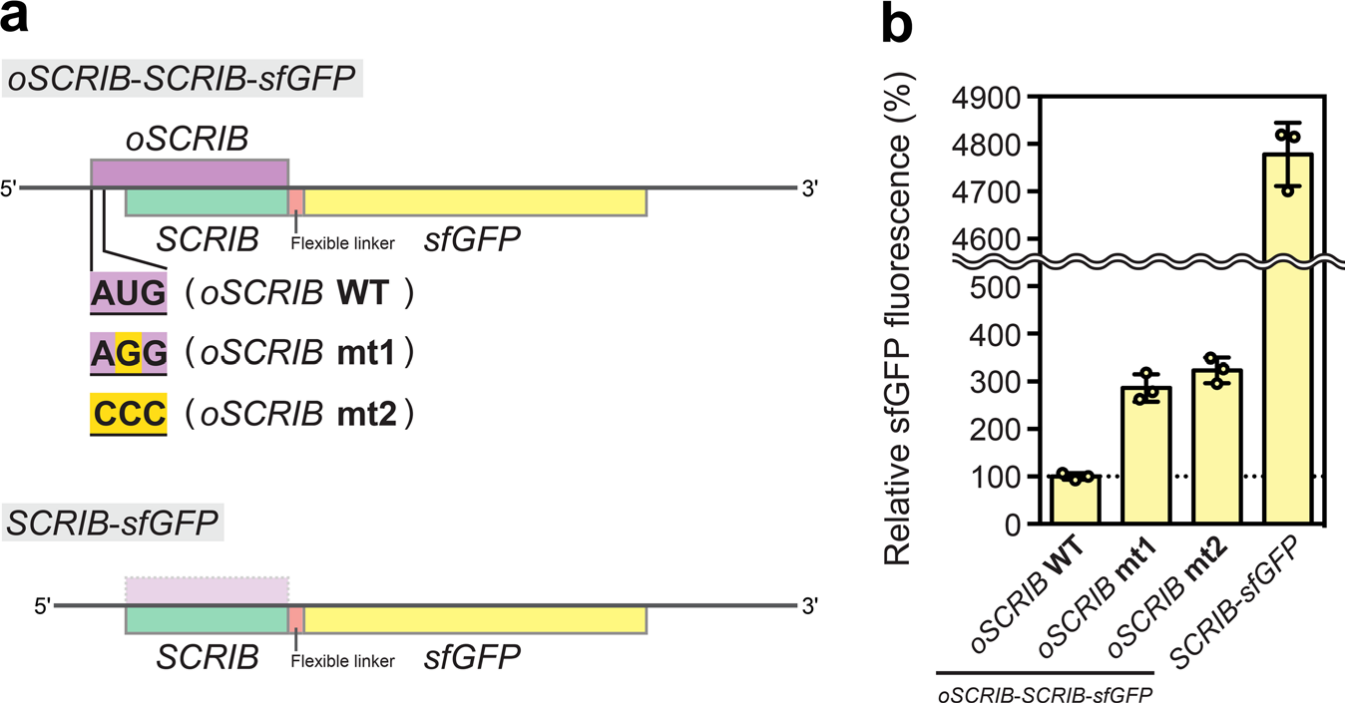
The functional role of *oSCRIB* in *SCRIB* translation. **a** The design of linear DNA fragments used as templates for in vitro transcription and translation. **b** The translational efficiency of *SCRIB* was quantified by the fluorescence intensity of the reporter protein sfGFP. Data are the mean ± SD of three independent reactions.

To investigate the functional role of *oSCRIB* in *SCRIB* translation, we employed an in vitro translation system based on eukaryotic 80 S ribosomes^51^. We first constructed linear DNA fragments that were used as templates for in vitro transcription and translation. The DNA fragments were constructed to code for a full-length *oSCRIB* and a partial *SCRIB* that was C-terminally truncated and fused to the reporter gene *sfGFP* through a flexible linker (*SCRIB*-*sfGFP*) (Fig. 6a). To assess the functionality of *oSCRIB*, we also mutated the translational start codon (AUG) of *oSCRIB* into AGG and CCC, both of which are not recognized as the start codons by eukaryotic ribosomes^51^. The DNA fragment encoding *SCRIB*–*sfGFP* alone was used as a positive control of in vitro translation. Then in vitro transcription and the subsequent translation were performed with the DNA fragments. The translational efficiency of *SCRIB* was quantified by the fluorescence intensity of the reporter protein sfGFP. The *oSCRIB*–*SCRIB*–*sfGFP* and *SCRIB*–*sfGFP* constructs showed that existence of the *oSCRIB* sequence drastically decreased the fluorescence intensity of sfGFP, indicating the inhibitory effect of the *oSCRIB* sequence on *SCRIB* translation (Fig. 6b). This was probably due to its extremely high GC base content (87%) (Supplementary Tables 1-2) and potential stem-loop GC base pairing, which leads to reduced translational efficiency^52^. Furthermore, disrupting the start codon (AUG) of *oSCRIB* by mutating it to AGG and CCC increased the fluorescence intensity of sfGFP, suggesting that abolishing the translatability of *oSCRIB* instead increased ribosomal translation of *SCRIB* (Fig. 6b). Therefore, our results confirmed that the newly discovered *oSCRIB* and its ribosomal translation constitutively limit the capacity of eukaryotic ribosomes to translate the downstream *SCRIB*.

## Discussion

We discovered a new small protein oSCRIB in human cancer proteomes using a proteogenomic approach. Importantly, *oSCRIB* translation by eukaryotic ribosomes occurred on a polycistronic mRNA encoding two adjacent protein-coding genes, *oSCRIB* and *SCRIB*, which correspond to oORF (or altORF^CDS^) and refCDS, respectively. The generated oSCRIB protein (i.e., an SEP), if bioactive, could directly impact cellular behavior^2,7,9^.

**Fig. 7:**
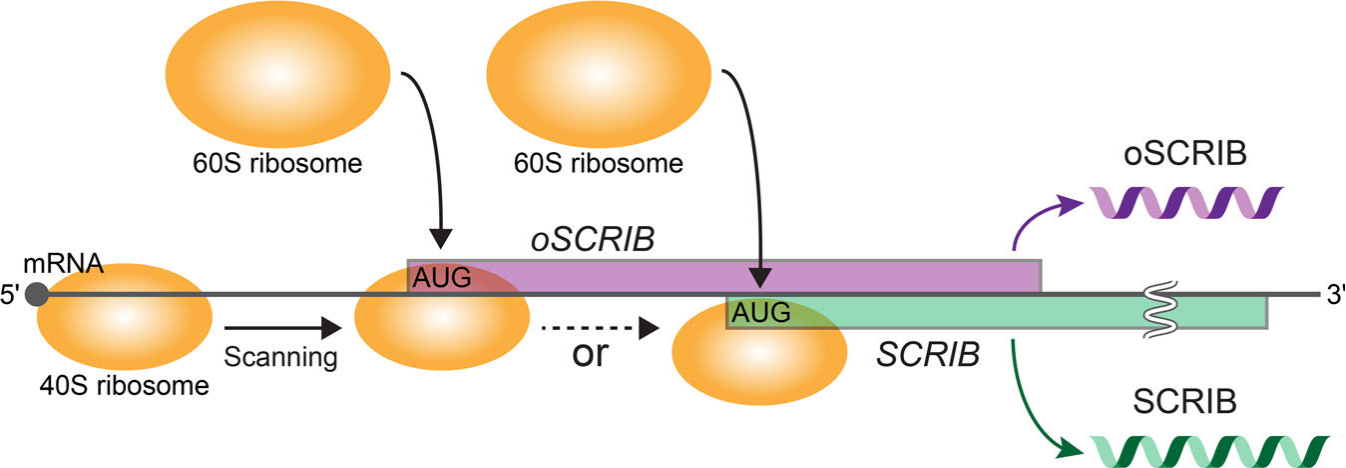
Proposed translational switch of the bicistronic *oSCRIB*–*SCRIB* gene pair in humans. Eukaryotic ribosome scanning (5′–3′) of the bicistronic mRNA for a translational start codon (AUG) and the subsequent translation initiation of *oSCRIB* can hinder that of the downstream out-of-frame tumor suppressor gene *SCRIB*, as supported by studies of oORFs^12,13,53^. *SCRIB* translation seems to be dependent on the frequency of leaky scanning^2,54^, whereby eukaryotic ribosomes bypass the start codon of *oSCRIB* and reach that of *SCRIB*.

The next important point to note is that the start codon of the downstream *SCRIB* is completely embedded in the coding region of *oSCRIB* (Fig. 2a). Hence, the translation initiation of *oSCRIB* through 5′ to 3′ scanning of the polycistronic mRNA by eukaryotic ribosomes could hinder that of the downstream out-of-frame *SCRIB* (Fig. 7), as strongly supported by previous studies on oORFs^12,13,53^. In other words, *SCRIB* translation appeared to be dependent on the frequency of leaky scanning^2,54^, whereby eukaryotic ribosomes bypass the start codon of *oSCRIB* and reach that of *SCRIB* (Fig. 7). Our biochemical analysis also supports these views as abolishing the translatability of *oSCRIB* by disrupting its start codon increased *SCRIB* translation (Fig. 6). Given that both oSCRIB and SCRIB proteins were detected in human cell and tissue proteomes (as described in the “Results” section), the existence of *oSCRIB* does not abolish the translatability of *SCRIB*, likely due to leaky scanning. Eukaryotic ribosomes tend to adopt a start codon surrounded by stronger sequence context or a Kozak sequence (e.g., ACCAUGG in human), and begin translation^55,56^. Given that *oSCRIB* lacks a purine (A or G) at position –3 relative to the start codon (AUG), its sequence context is assumed to be weaker than that of *SCRIB* (Fig. 2a). This disadvantaged sequence context of *oSCRIB* could permit leaky scanning of the polycistronic mRNA and subsequent translation initiation of *SCRIB* to some extent.

Recent work has provided strong evidence that altORF translation, which can arise from unconventional translation with an alternative eukaryotic-initiation factor 2 A in cancer, is an early and important event in oncogene expression and tumor formation^14^. In addition, translational dysregulation of altORF-dependent refCDS (or cancer driver gene) expression has been demonstrated to be important in tumor development and manipulation of immune-checkpoint proteins^13^. Similarly, our clinical proteomic data reanalysis also clarified translational dysregulation of the *oSCRIB*–*SCRIB* gene pair in human endometrial and breast carcinogenesis.

The translation product of *SCRIB* is known as a cell-polarity determinant and a large multi-domain scaffold POTSF protein for many key pathways such as antitumorigenic Hippo-YAP/TAZ, Ras/Raf/MEK/ERK (MAPK/ERK), and PI3K/Akt/mTOR and proapoptotic c-Myc-induced signaling pathways^22–29^. Hence, the regulatory role of *oSCRIB* in *SCRIB* translation is likely to be associated with maintaining cellular homeostasis in normal cells. Although further investigations are needed into *oSCRIB*-dependent *SCRIB* translation in humans and its biological contribution, our biochemical analysis suggested that the newly discovered *oSCRIB* and its translation constitutively limit the capacity of eukaryotic ribosomes to translate the downstream *SCRIB*. Therefore, it is reasonable to assume that the translatable *oSCRIB* is a *cis*-regulatory oORF that functions as a ribosomal roadblock and potentially provides a fail-safe mechanism to normal cells for nonexcessive downstream *SCRIB* expression. This limiting mechanism by *oSCRIB* region seemed to restrain the overexpression of *SCRIB* as a cancer-promoting POTSF not only in normal cells but also in cancer cells; however, our clinical proteomic data reanalysis indicated that it was dysregulated in cancer cells for *SCRIB* overexpression, resulting in their survival and proliferation. These results suggest that cancer cells weakened the effectiveness of the fail-safe mechanism, presumably by increasing the abundance of *oSCRIB*–*SCRIB* mRNA and the efficiency of ribosome loading onto the mRNA^23^. However, since our biochemical analysis clearly indicated that abolishing the translatability of *oSCRIB* results in *SCRIB* overexpression (Fig. 6), the existence of *oSCRIB* region is assumed to partially contribute to limit *SCRIB* expression in cancer cells and reduce malignant potential of cancer cells to some extent. In other words, lack of the *oSCRIB* region might further increase malignant potential of cancer cells. Thus, we anticipate that our findings will provide insight into research on cancer and associated biomarkers^57^. Taken together, the newly identified oORF, *oSCRIB*, is a promising translational regulatory element for *SCRIB* translation in the human genome. Additionally, the existence of the most prevalent sequence (GGACU) for the reversible epitranscriptomic m^6^A modification within oSCRIB-coding region (Fig. 2a) might represent a hidden layer of translational regulation exerted by the dynamic m^6^A modification^58,59^.

In this study, we newly constructed the custom sequence databases for SEP discovery. Although our databases consisted of millions of SEP candidates, only a limited number (–0.06%) of SEP candidates were detected in the publicly available high-quality proteomic data. This may imply the existence of SEPs expressed under specific cellular conditions. Thus, together with further validation of the probability-based identification of the remaining high-to-medium-confidence candidates, further proteomic analyses of cells/tissues treated with various stresses might be important in discovering a greater number of SEP candidates in the future.

In conclusion, our proteogenomics-driven gene discovery, which was supported by the integration of publicly available high-quality data from multishot deep proteomics and unbiased transcriptome annotation, proved its capability to mine hidden proteins again. Together with the data-driven multi-omics strategy, cell-free production of authentic proteins facilitated the rapid validation of probability-based SEP identification, thereby providing conclusive evidence of the SEP discovery. This workflow should ultimately lead to complete annotation of the human genome and improve our fundamental understanding of the “blueprint of life” in the future.

## Methods

### Transcriptomic data processing

Publicly available paired-end RNA-Seq data of polyadenylated mRNAs from several cancer cell lines (three biological replicates for each cell line) were downloaded as a single Sequence Read Archive (SRA) format file or a set of FASTQ format files as follows: the paired-end SRA format data of HeLa (1.57 × 10^8^ read pairs), MCF-7 (1.60 × 10^8^ read pairs), A549 (1.61 × 10^8^ read pairs), and HCT-116 (1.45 × 10^8^ read pairs) cells deposited in the NCBI BioProject (accession PRJNA523380) by the Cancer Cell Line Encyclopedia (CCLE)^60,61^; the paired-end FASTQ format data of HeLa (1.21 × 10^8^ and 1.18 × 10^8^ read pairs), MCF-7 (1.28 × 10^8^ and 1.32 × 10^8^ read pairs), A549 (7.11 × 10^7^ and 7.72 × 10^7^ read pairs), and HCT-116 (4.59 × 10^7^ and 4.59 × 10^7^ read pairs) cells deposited in the Encyclopedia of DNA Elements (ENCODE)^62^ database (https://www.encodeproject.org). The paired-end SRA format data were then converted to a set of FASTQ format files using a utility called fasterq-dump in NCBI SRA Toolkit 2.9.6-1^63^. Acquired raw paired-end reads in the FASTQ files were trimmed with Trim Galore! 0.6.2^64^, and the resultant trimmed reads were subjected to *de novo* assembly using Trinity 2.8.5^65^ with default settings. Possible ORFs within the assembled sequences and, additionally, human RefSeq Transcripts^30^ (NCBI RefSeq assembly accession GCF_000001405.39) were predicted by TransDecoder.LongOrfs 5.5.0^65^ with a parameter of the minimum codon length of 10, i.e., minimum protein length of nine amino acids. Next, all possible sORFs possibly encoding SEPs of 10–149 amino acids that start and stop with AUG and stop codons, respectively, were extracted using SeqKit 0.12.0^66^. The resulting datasets of sORFs from three replicates of each cell line were aggregated into a nonredundant dataset for each cell line. The translated nucleotide sequences of sORFs, i.e., the amino acid sequences of putative SEPs, were further searched against human RefSeq Proteins^30^ (NCBI RefSeq assembly accession GCF_000001405.39) using a stand-alone version of the BLAST program, BLAST + 2.9.0^67^. The resulting datasets, SEP sequences and their protein annotations by BLAST+, were combined with SeqKit, and only unannotated sequences were retained in each dataset (HeLa, MCF-7, A549, HCT-116, and RefSeq Transcripts). The dataset of RefSeq Transcripts was further integrated into that of each cell line and the resulting nonredundant datasets were used as the custom SEP sequence databases for HeLa, MCF-7, A549, and HCT-116 cells.

### DNA construction

Each DNA fragment that corresponded to *oSCRIB*, *uMKKS1*, *sfGFP*, and *oSCRIB*–*SCRIB*–*sfGFP* was produced and introduced into a pEX-A2J2 vector (Eurofins Genomics, Tokyo, Japan) by Eurofins Genomics (Supplementary Table 2). These DNA sequences were also confirmed by Eurofins Genomics. Then the coding regions with their 3′-UTR derived from the pEX-A2J2 vector were amplified by polymerase chain reaction (PCR) with Tks Gflex DNA polymerase (Takara Bio, Shiga, Japan) and primers listed in Supplementary Table 3 as follows: forward and reverse primers, Fw1-S and Rv1 for *oSCRIB* and *uMKKS1*, Fw1-G and Rv1 for *sfGFP*, Fw1-SG1 and Rv1 for *oSCRIB* (WT; ATG^start^)-*SCRIB*-*sfGFP*, Fw1-SG2 and Rv1 for *oSCRIB* (mt1; ATG^start^ to AGG)-*SCRIB*-*sfGFP*, Fw1-SG3 and Rv1 for *oSCRIB* (mt2; ATG^start^ to CCC)-*SCRIB*-*sfGFP*, and Fw1-SG4 and Rv1 for *SCRIB*–*sfGFP*. Each of the first PCR products was used as a template for a second PCR with primers listed in Supplementary Table 3 as follows: split forward primers, Fw2-E and Fw3-E (3 and 300 nM, respectively), and a nested reverse primer Rv2 (300 nM) for *oSCRIB*, *uMKKS1*, and *sfGFP*, and other split forward primers, Fw2-W and Fw3-W (3 and 300 nM, respectively), and the nested reverse primer Rv2 (300 nM) for *oSCRIB* (WT, mt1, and mt2)-*SCRIB*-*sfGFP* and *SCRIB*–*sfGFP*. The second PCR products were purified and concentrated with an Illustra GFX PCR DNA and gel band purification kit (GE Healthcare, Chalfont St. Giles, UK). To confirm the molecular size of the resulting DNA fragments, they were subjected to 1% (w/v) agarose gel electrophoresis and visualized under ultraviolet light on the gel stained with GelRed nucleic acid gel stain (FUJIFILM Wako Chemicals, Osaka, Japan). The DNA fragments were used for in vitro transcription and translation.

### In vitro transcription and translation

Bacterial translation coupled with transcription was performed in vitro with the PURE*frex* 2.0 system (GeneFrontier, Chiba, Japan). The reactions were initiated with the addition of 5 μL of DNA fragment (*oSCRIB*, *uMKKS1*, or *sfGFP*) into a 15-μL mixture of RNase-free water and PURE*frex* 2.0 Solution I, II, and III (GeneFrontier). The reaction mixtures were incubated at 37 °C for 5 h. Then the reaction mixtures were centrifuged at 20,400 × *g* for 20 min at 4 °C, and the resulting supernatants (soluble fractions) and pellets (insoluble fractions) were applied to SDS-PAGE. The gel was stained with Coomassie Brilliant Blue G-250. Eukaryotic translation was performed in vitro with the WEPRO7240H Expression Kit (CellFree Sciences, Matsuyama, Japan) as previously described^51^ with minor modifications. The reaction mixtures were then used to quantify the fluorescence intensity of the reporter protein sfGFP at excitation/emission wavelengths of 485/535 nm using a Wallac 1420 Multilabel Counter ARVO MX (PerkinElmer, Waltham, MA, USA).

### Protein extraction from HeLa cells

HeLa cells were grown and harvested as previously described^68^, and the collected cells were frozen at −80 °C until subsequent use. The frozen cells were thawed on ice and lysed by ultrasonic treatment in a solution containing 50 mM sodium phosphate buffer (pH 7.9) and 500 mM NaCl. The cell extracts were applied to SDS-PAGE for protein separation. The gel was stained with Coomassie Brilliant Blue G-250.

### Sample preparation for LC–MS/MS analysis

The protein bands that corresponded to in vitro-synthesized proteins (oSCRIB and uMKKS1) and endogenous HeLa proteins of approximately 10–15 kDa, which included oSCRIB protein (12 kDa), were excised from the SDS-PAGE gels. The gel slices were destained and treated with 50 mM dithiothreitol followed by 100 mM sodium iodoacetate for protein reduction and alkylation (carboxymethylation), respectively. Then the gel slices were washed with water. The proteins in the gel were digested with *N*‐tosyl-l-phenylalanine chloromethyl ketone‐treated trypsin (Worthington Biochemical, Freehold, NJ, USA) in a solution containing 20 mM Tris-HCl buffer (pH 8.0) and 0.05% (w/v) *n*-dodecyl-β-d-maltoside at 37 °C for 16 h. The resultant peptides were analyzed by LC-MS/MS.

### LC–MS/MS analysis

The samples were applied to a packed nanocapillary C18 column (NTCC-360/75-3-105, 0.075 × 105-mm column, particle size: 3 μm; Nikkyo Technos, Tokyo, Japan) for LC–MS/MS analysis with an Easy-nLC 1000 liquid chromatography system (Thermo Fisher Scientific, Waltham, MA, USA). Two types of eluent were used in the column: eluent A consisted of water containing 0.1% (v/v) formic acid and eluent B consisted of acetonitrile containing 0.1% (v/v) formic acid. The column was kept at room temperature, and eluent A was applied as an initial eluent to the column at a flow rate of 300 nL/min. Elution was performed by increasing the proportion of eluent B to eluent A from 0% to 100% over 12 min (35% at 10 min and 100% at 12 min). The last condition was maintained for 8 min. The Q Exactive hybrid Quadrupole-Orbitrap mass spectrometer^46^ (Thermo Fisher Scientific) was used to analyze the eluate in electrospray-ionization (ESI) positive-ion mode. The MS/MS spectra were obtained by the mass spectrometer that was operated in DDA mode to automatically alternate between a full scan of precursor ions (*m/z* 300–2000) in the Orbitrap (resolution, 70,000; automatic gain-control target, 3,000,000; maximum injection time, 60 ms) and subsequent HCD–MS/MS scans of the 10 most abundant (top 10) precursor ions (*m/z* 200–2000) in the Orbitrap (resolution, 17,500; automatic gain-control target, 500,000; maximum injection time, 100 ms; isolation window, 4.0 *m/z*; normalized collision energy, 30%).

For PRM analysis, sample elution from the column was performed by increasing the proportion of eluent B to eluent A from 0% to 100% over 230 min (1% at 1 s, 2% at 10 min, 10% at 120 min, 20% at 180 min, 40% at 220 min, and 100% at 230 min). The last condition was maintained for 20 min. The mass spectrometer was used to analyze the eluate in ESI positive-ion mode. The MS/MS spectra were obtained by the mass spectrometer that was operated in the PRM mode to perform the HCD–MS/MS scans of selected precursor ions, [M + 3H]^3+^ of TEPRPPAPSPPSAAAGAR (*m/z* 577.30419), [M + 3H]^3+^ and [M + 4H]^4+^ of AAHPHHAQVHPAVALQPAR (*m/z* 670.02939 and 502.77386, respectively), [M + 2H]^2+^ of GVGGQAALFAAGR (*m/z* 587.82000), [M + 2H]^2+^ of AGGDLPLQPQPGGAAAR (*m/z* 788.41553), and [M + 2H]^2+^ and [M + 3H]^3+^ of AAQAFFPAAELAQAGPER (*m/z* 922.96812 and 615.64784, respectively), in the Orbitrap (resolution, 140,000; automatic gain-control target, 3,000,000; maximum injection time, 500 ms; isolation window, 0.8 *m/z*; normalized collision energy, 30%).

### Proteomic data processing

All raw MS/MS files, including the one analyzed above and the publicly available proteome datasets of human cell lines (HeLa, MCF-7, A549, and HCT-116; two biological replicates for each cell line) deposited in the ProteomeXchange^35^ (dataset identifier PXD004452^34^) and human primary tumor and adjacent normal tissues (endometria^49^ and breasts^50^) deposited in the Clinical Proteomic Tumor Analysis Consortium (CPTAC) Data Portal (https://cptac-data-portal.georgetown.edu/study-summary/S053, https://cptac-data-portal.georgetown.edu/study-summary/S060), were converted into MGF files using Thermo Proteome Discoverer 2.2.0.388 (Thermo Fisher Scientific). The publicly available proteomic data files were first processed to recalibrate precursor masses and then converted into MGF files with Thermo Proteome Discoverer. The MGF files were submitted to an in-house Mascot Server 2.7.0 (Matrix Science, London, UK) and subjected to PSM searches by the Mascot algorithm through Thermo Proteome Discoverer with target- and decoy-sequence databases and the following parameters: variable modifications, acetyl (protein N-term), oxidation (M), and Gln to pyro-Glu conversion (N-term Q); static modification, carboxymethyl (C) for the samples analyzed above or carbamidomethyl (C) for the publicly available proteome datasets and TMT6plex (N-term and K) for the publicly available proteome datasets of human primary tumor/normal adjacent tissues; maximum of three missed cleavages by trypsin digestion; precursor mass tolerance of 15 ppm; fragment mass tolerance of 30 mmu. The following target-sequence databases and their decoy-sequence databases generated by reversing the target sequences with Thermo Proteome Discoverer were each used in separate PSM searches for target decoy-based FDR estimation^37,38^. For proteogenomic data mining, the two different target databases in parallel, i.e., the combination of human RefSeq Proteins and the custom SEP-sequence database (for HeLa, MCF-7, A549, or HCT-116) as constructed above were used; for the detection of the in vitro-synthesized proteins, the target database that consists of the protein sequences of oSCRIB and uMKKS1 and the whole-protein sequences of *Escherichia coli* BL21 (DE3) (NCBI GenBank accession CP001509, version CP001509.3^69^) were used; for the detection of endogenous HeLa proteins of approximately 10–15 kDa, the target database that consists of the protein sequences of oSCRIB and uMKKS1 and the previously annotated SEP sequences (–150 amino acids) collected from human RefSeq Proteins was used; and for reanalysis of clinical human proteomic data (TMT-based quantitative proteomic data) on human primary tumor and adjacent normal tissues (endometria and breasts), the target database that consists of the protein sequences of uSCRIB and oSCRIB and human RefSeq Proteins (including SCRIB) was used. The search results were filtered by the Percolator algorithm^37,38^ to maintain an estimated target decoy-based FDR of 1% at the peptide and protein levels. The identified peptides/proteins were further searched against the universal protein resource (UniProt) database (UniProt Knowledgebase). A peptide-centric search of the oSCRIB-derived peptides (AGGDLPLQPQPGGAAAR and AAQAFFPAAELAQAGPER) and the uMKKS1-derived peptide (NDDIPEQDSLGLSNLQK) from the publicly available proteomic data (MGF format) was executed by a stand-alone version (1.6.0) of the PepQuery program^39^ with default parameters (reference protein database, human RefSeq Proteins; variable modification, oxidation (M); static modification, carbamidomethyl (C); maximum of two missed cleavages by trypsin digestion; precursor mass tolerance of 10 ppm; fragment mass tolerance of 600 mmu; and scoring algorithm, Hyperscore). Manual inspection of MS/MS spectra was performed with Thermo Proteome Discoverer and Thermo Xcalibur Qual Browser 3.1.66.10 (Thermo Fisher Scientific). The results were then illustrated using an integrative proteomics data viewer PDV 1.6.0^70^. Mapping of DNA sequences corresponding to peptides/proteins of interest onto the human genome was conducted with an integrative genomics viewer IGV 2.8.0^71^.

### Reporting summary

Further information on research design is available in the Nature Research Reporting Summary linked to this article.

## Supplementary information

Supplementary Information

Description of Additional Supplementary Files

Supplementary Data 1

Reporting Summary

## Data Availability

All data supporting the findings of this study are available within the paper and its supplementary information file. Uncropped and unedited gel images are included in Supplementary Figure 1. Source data for graphs are included in Supplementary Data 1. The protein-sequence data reported in this paper will appear in the UniProt Knowledgebase under the accession number C0HLS1 for oSCRIB in humans (*Homo sapiens*). The raw mass spectrometric data and Mascot-related files have been deposited to the ProteomeXchange Consortium^35^ via the PRIDE partner repository^72^ with the dataset identifier PXD027841 and 10.6019/PXD027841.
