# Supplementary material for: Discovery of a small protein-encoding cis-regulatory overlapping gene of the tumor suppressor gene Scribble in humans
Source: Commun Biol. 2021 Sep 17;4:1098. doi: 10.1038/s42003-021-02619-8 (PMC8448870; doi:10.1038/s42003-021-02619-8)
Supplement: Supplementary file 1 — Supplementary Information [file 42003_2021_2619_MOESM1_ESM.pdf]

Supplementary information

Discovery of a small protein-encoding *cis*-regulatory overlapping gene of the tumor suppressor gene *Scribble* in humans

Yuhta Nomura[1*] and Naoshi Dohmae[1*]

[1]Biomolecular Characterization Unit, RIKEN Center for Sustainable Resource Science, 2-1 Hirosawa, Wako, Saitama, 351-0198, Japan. [*]Correspondence and requests for materials should be addressed to Y. N. (email: yuta.nomura@riken.jp) or to N. D. (email: dohmae@riken.jp)

Supplementary Table 1. The nucleotide and protein sequences of human *oSCRIB* and *uMKKS1*

No	Name	Sequence (5' to 3')*
1	*oSCRIB*	ATGCGGACTGAGCCCCGCCCCCCGGCCCCGAGCCCGCCGAGCGCCGCCGCCGGA<br>GCCCGCGCCGCCCACCCGCACCATGCTCAAGTGCATCCCGCTGTGGCGCTGCAAC<br>CGGCACGTGGAGTCGGTGGACAAGCGGCACTGTTCGCTGCAGGCCGTGCCGGAG<br>GAGATCTACCGCTACAGCCGCAGCCTGGAGGAGCTGCTGCTCGACGCCAACCAGC<br>TGCGCGAGCTGCCCAAGCCTTTTTTCCGGCTGCTGAACTTGCGCAAGCTGGGCCTG<br>AGCGACAACGAGATCCAGCGGTTGCCTCCCGAGGTGGCCAACTTCATGCAGCTGG<br>TGGAGCTGGACGTGTCCCGGAACGATATCCCTGA<br><br>Translation product:<br><br>MRTEPRPPAPSPPSAAAGARAAHPHHAQVHPAVALQPARGVGGQAALFAAGRAGGD<br>LPLQPQPGGAAARRQPAARAAQAFFPAAELAQAGPERQRDPAVASRGGQLHAAGGA<br>GRVPERYP
2	*uMKKS1*	ATGAGCCTTCGGAACTTGTGGAGAGACTACAAAGTTTTGGTTGTTATGGTCCCTTT<br>AGTTGGGCTCATACATTTGGGGTGGTACAGAATCAAAAGCAGCCCTGTTTTCCAA<br>ATACCTAAAAACGACGACATTCCTGAGCAAGATAGTCTGGGACTTTCAAATCTTC<br>AGAAGAGCCAAATCCAGGGGAAGTAG<br><br>Translation product:<br><br>MSLRNLWRDYKVLVVMVPLVGLIHLGWYRIKSSPVFQIPKNDDIPEQDSLGLSNLQK<br>SQIQGK

*Boxes indicate the translational start and stop codons of each protein.

Supplementary Table 2. List of plasmids

No	Name	Sequence (5' to 3')*
1	pEX-A2J2-*oSCRIB*	AGATATCCATATGTTTACTAGTAACAATGGGACATCATCATCATCATCACGATTACGA<br>CATCCCAACGACCGAAAACCTGTATTTTCAGGGCAAGATGAGAACCGAACCTAGACCA<br>CCTGCACCATCCCCACCAAGCGCAGCTGCTGGTGCTAGAGCTGCTCACCCACATCATG<br>CACAAGTGCACCCAGCTGTGGCACTCCAACCAGCAAGAGGTGTCGGCGGGCAAGCCG<br>CCTTGTTTGCTGCCGGCAGAGCTGGAGGAGATCTTCCACTCCAACCACAACCAGGCGG<br>TGCTGCTGCTCGAAGACAACCAGCAGCAAGGGCTGCTCAAGCTTTCTTCCCAGCTGCT<br>GAGCTGGCTCAAGCTGGCCCTGAGCGTCAACGCGACCCAGCTGTCGCTTCGCGCGGAG<br>GCCAACTGCACGCTGCTGGAGGCGCTGGACGTGTTCCAGAGCGCTACCCATGAGTCGA<br>CTTTCCCGGGTAACTAACTAAGGATCCTTTGAGCTCGGTACCGATATCAATGCCGTGAG<br>CACAAAAGCGACCATAGTCGGGATTTACCCGCTTATCTGCGGAAGGTCATTGGGAAC<br>GCATCTTCCACAAATGGGCTGAGCGCAAAAATGCCGAACGTATCTGGCTGACCTATGC<br>CATTCCGTGGGAACGACACTACTTTTCAATTGGTGCACTCTCAGTACAATCTGCTCTGA<br>TGCCGCATAGTTAAGCCAGCCCCGACACCCGCCAACACCCGCTGACGCGCCCTGACGG<br>GCTTGTCTGCTCCCGGCATCCGCTTACAGACAAGCTGTGACCGTCTCCGGAAAATCAA<br>AGGATCTTCTTGAGATCCTTTTTTTGAGCTGCATGTGTCAGAGGTTTTCACCGTCATCA<br>CCGAAACGCGCGAGACGAAAGGGCCTCGTGATACGCCTATTTTTATAGGTTAATGTCA<br>TGATAATAATGGTTTCTTAGACGTCAGGTGGCACTTTTCGGGGAAATGTGCGCGGAAC<br>CCCTATTTGTTTATTTTTCTAAATACATTCAAATATGTATCCGCTCATGAGACAATAAC<br>CCTGATAAATGCTTCAATAATATTGAAAAAGGAAGAGTATGAGTATTCAACATTTCCG<br>TGTCGCCCTTATTCCCTTTTTTGCGGCATTTTGCCTTCCTGTTTTTGCTCACCCAGAAAC<br>GCTGGTGAAAGTAAAAGATGCTGAAGATCAGTTGGGTGCACGAGTGGGTTACATCGA<br>ACTGGATCTCAACAGCGGTAAGATCCTTGAGAGTTTTCGCCCCGAAGAACGTTTTCCA<br>ATGATGAGCACTTTTAAAGTTCTGCTATGTGGCGCGGTATTATCCCGTATTGACGCCGG<br>GCAAGAGCAACTCGGTCGCCGCATACACTATTCTCAGAATGACTTGGTTGAGTACTCA<br>CCAGTCACAGAAAAGCATCTTACGGATGGCATGACAGTAAGAGAATTATGCAGTGCTG<br>CCATAACCATGAGTGATAACACTGCGGCCAACTTACTTCTGACAACGATCGGAGGACC<br>GAAGGAGCTAACCGCTTTTTTGCACAACATGGGGGATCATGTAACTCGCCTTGATCGTT<br>GGGAACCGGAGCTGAATGAAGCCATACCAAACGACGAGCGTGACACCACGATGCCTG<br>TAGCAATGGCAACAACGTTGCGCAAACTATTAACTGGCGAACTACTTACTCTAGCTTC<br>CCGGCAACAATTAATAGACTGGATGGAGGCGGATAAAGTTGCAGGACCACTTCTGCGC<br>TCGGCCCTTCCGGCTGGCTGGTTTATTGCTGATAAATCTGGAGCCGGTGAGCGTGGGA<br>GTCGCGGTATCATTGCAGCACTGGGGCCAGATGGTAAGCCCTCCCGTATCGTAGTTAT<br>CTACACGACGGGGAGTCAGGCAACTATGGATGAACGAAATAGACAGATCGCTGAGAT<br>AGGTGCCTCACTGATTAAGCATTGGTAACTGTCAGACCAAGTTTACTCATATATACTTT<br>AGATTGATTTAAAACTTCATTTTTAATTTAAAAGGATCTAGGTGAAGATCCTTTTTGAT<br>AATCTCATGACCAAAATCCCTTAACGTGAGTTTTCGTTCCACTGAGCGTCAGACCCCGT<br>AGAAAAGATCAAAGGATCTTCTTGAGATCCTTTTTTTCTGCGCGTAATCTGCTGCTTGC<br>AAACAAAAAAACCACCGCTACCAGCGGTGGTTTGTTTGCCGGATCAAGAGCTACCAAC<br>TCTTTTTCCGAAGGTAACTGGCTTCAGCAGAGCGCAGATACCAAATACTGTTCTTCTAG<br>TGTAGCCGTAGTTAGGCCACCACTTCAAGAACTCTGTAGCACCGCCTACATACCTCGCT<br>CTGCTAATCCTGTTACCAGTGGCTGCTGCCAGTGGCGATAAGTCGTGTCTTACCGGGTT<br>GGACTCAAGACGATAGTTACCGGATAAGGCGCAGCGGTCGGGCTGAACGGGGGGTTC<br>GTGCACACAGCCCAGCTTGGAGCGAACGACCTACACCGAACTGAGATACCTACAGCGT<br>GAGCTATGAGAAAGCGCCACGCTTCCCGAAGGGAGAAAGGCGGACAGGTATCCGGTA<br>AGCGGCAGGGTCGGAACAGGAGAGCGCACGAGGGAGCTTCCAGGGGGAAACGCCTGG<br>TATCTTTATAGTCCTGTCGGGTTTCGCCACCTCTGACTTGAGCGTCGATTTTTGTGATGC<br>TCGTCAGGGGGGCGGAGCCTATGGAAAAACGCCAGCAACGCGGCCTTTTTACGGTTCC<br>TGGCCTTTTGCTGGCCTTTTGCTCACATGTTCTTTCCTGCGTTATCCCCTGATTCTGTGG<br>ATAACCGTATTACCGCCTTTGAGTGAGCTGATACCGCTCGCCGCAGCCGAACGACCGA<br>GCGCAGCGAGTCAGTGAGCGAGGAAGCGGAAGAGCAAATAATAAAAAAGCCGGATTA<br>ATAATCTGGCTTTTTATATTCTCTGCCCAATACGCAAACCGCCTCTCCCCGCGCGTTGG<br>CCGATTCATTAATGCAGCTGGCACGACAGGCAAAGACTTCTACGGTTGGGCAATCGAA<br>CGCCACTATTCAGCGCATGACCTGTTTAAGGCGCATAGCTTTGATCATGCCGGTAGAG<br>CACGTTGGGAAGGACATCGTTGGGCACATCGTGAACATGCCAACATGTTTTGGGCGCA<br>TGAAGGCATTTGGCGTATCCACTGACT

*Boxes indicate the translational start and stop codons of the protein (oSCRIB). Underlines indicate the primer annealing sites. Gray highlight indicates the pEX-A2J2 vector sequence.

Supplementary Table 2. List of plasmids (continued)

No	Name	Sequence (5' to 3')*
2	pEX-A2J2-*uMKKS1*	AGATATCCATATGTTTACTAGTAACAATGGGACATCATCATCATCATCACGATTACGA<br>CATCCCAACGACCGAAAACCTGTATTTTCAGGGCAAGATGAGCCTGAGAAACTTATGG<br>AGGGACTACAAGGTCCTGGTAGTGATGGTCCCGCTCGTGGGCCTCATTCACTTGGGAT<br>GGTATCGCATAAAATCATCCCCCGTTTTCCAGATCCCAAAAAACGACGACATCCCTGA<br>ACAGGACAGTCTAGGGCTCTCTAATTTGCAAAAGTCGCAAATCCAAGGTAAGTGAGTC<br>GACTTTCCCGGGTAACTAACTAAGGATCCTTTGAGCTCGGTACCGATATCAATGCCGTG<br>AGCACAAAAGCGACCATAGTCGGGATTTTACCCGCTTATCTGCGGAAGGTCATTGGGA<br>ACGCATCTTCCACAAATGGGCTGAGCGCAAAAATGCCGAACGTATCTGGCTGACCTAT<br>GCCATTCCGTGGGAACGACACTACTTTTCAATTGGTGCACTCTCAGTACAATCTGCTCT<br>GATGCCGCATAGTTAAGCCAGCCCCGACACCCGCCAACACCCGCTGACGCGCCCTGAC<br>GGGCTTGTCTGCTCCCGGCATCCGCTTACAGACAAGCTGTGACCGTCTCCGGAAAATC<br>AAAGGATCTTCTTGAGATCCTTTTTTTGAGCTGCATGTGTCAGAGGTTTTCACCGTCAT<br>CACCGAAACGCGCGAGACGAAAGGGCCTCGTGATACGCCTATTTTTATAGGTTAATGT<br>CATGATAATAATGGTTTCTTAGACGTCAGGTGGCACTTTTCGGGGAAATGTGCGCGGA<br>ACCCCTATTTGTTTATTTTTCTAAATACATTCAAATATGTATCCGCTCATGAGACAATA<br>ACCCTGATAAATGCTTCAATAATATTGAAAAAGGAAGAGTATGAGTATTCAACATTTC<br>CGTGTCGCCCTTATTCCCTTTTTTGCGGCATTTTGCCTTCCTGTTTTTGCTCACCCAGAA<br>ACGCTGGTGAAAGTAAAAGATGCTGAAGATCAGTTGGGTGCACGAGTGGGTTACATCG<br>AACTGGATCTCAACAGCGGTAAGATCCTTGAGAGTTTTCGCCCCGAAGAACGTTTTCC<br>AATGATGAGCACTTTTAAAGTTCTGCTATGTGGCGCGGTATTATCCCGTATTGACGCCG<br>GGCAAGAGCAACTCGGTCGCCGCATACACTATTCTCAGAATGACTTGGTTGAGTACTC<br>ACCAGTCACAGAAAAGCATCTTACGGATGGCATGACAGTAAGAGAATTATGCAGTGCT<br>GCCATAACCATGAGTGATAACACTGCGGCCAACTTACTTCTGACAACGATCGGAGGAC<br>CGAAGGAGCTAACCGCTTTTTTGCACAACATGGGGGATCATGTAACTCGCCTTGATCG<br>TTGGGAACCGGAGCTGAATGAAGCCATACCAAACGACGAGCGTGACACCACGATGCC<br>TGTAGCAATGGCAACAACGTTGCGCAAACTATTAACTGGCGAACTACTTACTCTAGCT<br>TCCCGGCAACAATTAATAGACTGGATGGAGGCGGATAAAGTTGCAGGACCACTTCTGC<br>GCTCGGCCCTTCCGGCTGGCTGGTTTATTGCTGATAAATCTGGAGCCGGTGAGCGTGG<br>GAGTCGCGGTATCATTGCAGCACTGGGGCCAGATGGTAAGCCCTCCCGTATCGTAGTT<br>ATCTACACGACGGGGAGTCAGGCAACTATGGATGAACGAAATAGACAGATCGCTGAG<br>ATAGGTGCCTCACTGATTAAGCATTGGTAACTGTCAGACCAAGTTTACTCATATATACT<br>TTAGATTGATTTAAAACTTCATTTTTAATTTAAAAGGATCTAGGTGAAGATCCTTTTTG<br>ATAATCTCATGACCAAAATCCCTTAACGTGAGTTTTCGTTCCACTGAGCGTCAGACCCC<br>GTAGAAAAGATCAAAGGATCTTCTTGAGATCCTTTTTTTCTGCGCGTAATCTGCTGCTT<br>GCAAACAAAAAAACCACCGCTACCAGCGGTGGTTTGTTTGCCGGATCAAGAGCTACCA<br>ACTCTTTTTCCGAAGGTAACTGGCTTCAGCAGAGCGCAGATACCAAATACTGTTCTTCT<br>AGTGTAGCCGTAGTTAGGCCACCACTTCAAGAACTCTGTAGCACCGCCTACATACCTC<br>GCTCTGCTAATCCTGTTACCAGTGGCTGCTGCCAGTGGCGATAAGTCGTGTCTTACCGG<br>GTTGGACTCAAGACGATAGTTACCGGATAAGGCGCAGCGGTCGGGCTGAACGGGGGG<br>TTCGTGCACACAGCCCAGCTTGGAGCGAACGACCTACACCGAACTGAGATACCTACAG<br>CGTGAGCTATGAGAAAGCGCCACGCTTCCCGAAGGGAGAAAGGCGGACAGGTATCCG<br>GTAAGCGGCAGGGTCGGAACAGGAGAGCGCACGAGGGAGCTTCCAGGGGGAAACGCC<br>TGGTATCTTTATAGTCCTGTCGGGTTTCGCCACCTCTGACTTGAGCGTCGATTTTTGTGA<br>TGCTCGTCAGGGGGGCGGAGCCTATGGAAAAACGCCAGCAACGCGGCCTTTTTACGGT<br>TCCTGGCCTTTTGCTGGCCTTTTGCTCACATGTTCTTTCCTGCGTTATCCCCTGATTCTGT<br>GGATAACCGTATTACCGCCTTTGAGTGAGCTGATACCGCTCGCCGCAGCCGAACGACC<br>GAGCGCAGCGAGTCAGTGAGCGAGGAAGCGGAAGAGCAAATAATAAAAAAGCCGGA<br>TTAATAATCTGGCTTTTTATATTCTCTGCCCAATACGCAAACCGCCTCTCCCCGCGCGTT<br>GGCCGATTCATTAATGCAGCTGGCACGACAGGCAAAGACTTCTACGGTTGGGCAATCG<br>AACGCCACTATTCAGCGCATGACCTGTTTAAGGCGCATAGCTTTGATCATGCCGGTAG<br>AGCACGTTGGGAAGGACATCGTTGGGCACATCGTGAACATGCCAACATGTTTTGGGCG<br>CATGAAGGCATTTGGCGTATCCACTGACT

*Boxes indicate the translational start and stop codons of the protein (uMKKS1). Underlines indicate the primer annealing sites. Gray highlight indicates the pEX-A2J2 vector sequence.

Supplementary Table 2. List of plasmids (continued)

No	Name	Sequence (5' to 3')*
3	pEX-A2J2-*sfGFP*	AGATATCCATATGTTTACTAGTAACAATGTCTAAGGGAGAAGAACTGTTCACCGGCGT<br>CGTTCCGATTCTCGTCGAACTAGACGGCGATGTGAATGGCCATAAGTTCTCTGTGCGA<br>GGAGAAGGGGAGGGAGATGCCACCAACGGTAAACTCACCCTGAAATTCATCTGTACG<br>ACGGGGAAGTTACCAGTGCCGTGGCCTACCCTTGTTACTACGTTAACGTACGGGGTGC<br>AATGTTTTTCACGCTACCCCGACCACATGAAACGACACGATTTCTTTAAGTCGGCAATG<br>CCGGAGGGCTACGTGCAGGAACGTACGATCTCCTTCAAGGACGACGGAACCTACAAG<br>ACACGCGCGGAGGTCAAGTTCGAAGGTGATACCTTGGTCAACAGGATAGAACTGAAG<br>GGGATCGATTTCAAGGAGGACGGAAACATCCTAGGGCATAAGCTGGAGTACAATTTCA<br>ATAGTCATAACGTTTACATTACAGCAGACAAGCAAAAGAATGGGATTAAGGCCAATTT<br>CAAGATCCGGCATAATGTGGAAGACGGCTCCGTTCAACTAGCGGATCACTATCAACAG<br>AACACACCAATCGGCGACGGACCAGTCCTTCTACCAGACAATCATTATCTTAGCACCC<br>AAAGTGTTCTGTCGAAGGACCCGAACGAGAAGAGGGATCACATGGTTTTGTTGGAATT<br>TGTCACGGCAGCCGGAATCACTCATGGCATGGATGAATTGTATAAGGGCAGCCACCAC<br>CACCACCACCATTGAGTCGACTTTCCCGGGTAACTAACTAAGGATCCTTTGAGCTCGG<br>TACCGATATCAATGCCGTGAGCACAAAAGCGACCATAGTCGGGATTTTACCCGCTTAT<br>CTGCGGAAGGTCATTGGGAACGCATCTTCCACAAATGGGCTGAGCGCAAAAATGCCGA<br>ACGTATCTGGCTGACCTATGCCATTCCGTGGGAACGACACTACTTTTCAATTGGTGCAC<br>TCTCAGTACAATCTGCTCTGATGCCGCATAGTTAAGCCAGCCCCGACACCCGCCAACA<br>CCCGCTGACGCGCCCTGACGGGCTTGTCTGCTCCCGGCATCCGCTTACAGACAAGCTGT<br>GACCGTCTCCGGAAAATCAAAGGATCTTCTTGAGATCCTTTTTTTGAGCTGCATGTGTC<br>AGAGGTTTTCACCGTCATCACCGAAACGCGCGAGACGAAAGGGCCTCGTGATACGCCT<br>ATTTTTATAGGTTAATGTCATGATAATAATGGTTTCTTAGACGTCAGGTGGCACTTTTC<br>GGGGAAATGTGCGCGGAACCCCTATTTGTTTATTTTTCTAAATACATTCAAATATGTAT<br>CCGCTCATGAGACAATAACCCTGATAAATGCTTCAATAATATTGAAAAAGGAAGAGTA<br>TGAGTATTCAACATTTCCGTGTCGCCCTTATTCCCTTTTTTGCGGCATTTTGCCTTCCTG<br>TTTTTGCTCACCCAGAAACGCTGGTGAAAGTAAAAGATGCTGAAGATCAGTTGGGTGC<br>ACGAGTGGGTTACATCGAACTGGATCTCAACAGCGGTAAGATCCTTGAGAGTTTTCGC<br>CCCGAAGAACGTTTTCCAATGATGAGCACTTTTAAAGTTCTGCTATGTGGCGCGGTATT<br>ATCCCGTATTGACGCCGGGCAAGAGCAACTCGGTCGCCGCATACACTATTCTCAGAAT<br>GACTTGGTTGAGTACTCACCAGTCACAGAAAAGCATCTTACGGATGGCATGACAGTAA<br>GAGAATTATGCAGTGCTGCCATAACCATGAGTGATAACACTGCGGCCAACTTACTTCT<br>GACAACGATCGGAGGACCGAAGGAGCTAACCGCTTTTTTGCACAACATGGGGGATCAT<br>GTAACTCGCCTTGATCGTTGGGAACCGGAGCTGAATGAAGCCATACCAAACGACGAGC<br>GTGACACCACGATGCCTGTAGCAATGGCAACAACGTTGCGCAAACTATTAACTGGCGA<br>ACTACTTACTCTAGCTTCCCGGCAACAATTAATAGACTGGATGGAGGCGGATAAAGTT<br>GCAGGACCACTTCTGCGCTCGGCCCTTCCGGCTGGCTGGTTTATTGCTGATAAATCTGG<br>AGCCGGTGAGCGTGGGAGTCGCGGTATCATTGCAGCACTGGGGCCAGATGGTAAGCCC<br>TCCCGTATCGTAGTTATCTACACGACGGGGAGTCAGGCAACTATGGATGAACGAAATA<br>GACAGATCGCTGAGATAGGTGCCTCACTGATTAAGCATTGGTAACTGTCAGACCAAGT<br>TTACTCATATATACTTTAGATTGATTTAAAACTTCATTTTTAATTTAAAAGGATCTAGGT<br>GAAGATCCTTTTTGATAATCTCATGACCAAAATCCCTTAACGTGAGTTTTCGTTCCACT<br>GAGCGTCAGACCCCGTAGAAAAGATCAAAGGATCTTCTTGAGATCCTTTTTTTCTGCGC<br>GTAATCTGCTGCTTGCAAACAAAAAAACCACCGCTACCAGCGGTGGTTTGTTTGCCGG<br>ATCAAGAGCTACCAACTCTTTTTCCGAAGGTAACTGGCTTCAGCAGAGCGCAGATACC<br>AAATACTGTTCTTCTAGTGTAGCCGTAGTTAGGCCACCACTTCAAGAACTCTGTAGCAC<br>CGCCTACATACCTCGCTCTGCTAATCCTGTTACCAGTGGCTGCTGCCAGTGGCGATAAG<br>TCGTGTCTTACCGGGTTGGACTCAAGACGATAGTTACCGGATAAGGCGCAGCGGTCGG<br>GCTGAACGGGGGGTTCGTGCACACAGCCCAGCTTGGAGCGAACGACCTACACCGAACT<br>GAGATACCTACAGCGTGAGCTATGAGAAAGCGCCACGCTTCCCGAAGGGAGAAAGGC<br>GGACAGGTATCCGGTAAGCGGCAGGGTCGGAACAGGAGAGCGCACGAGGGAGCTTCC<br>AGGGGGAAACGCCTGGTATCTTTATAGTCCTGTCGGGTTTCGCCACCTCTGACTTGAGC<br>GTCGATTTTTGTGATGCTCGTCAGGGGGGCGGAGCCTATGGAAAAACGCCAGCAACGC<br>GGCCTTTTTACGGTTCCTGGCCTTTTGCTGGCCTTTTGCTCACATGTTCTTTCCTGCGTT<br>ATCCCCTGATTCTGTGGATAACCGTATTACCGCCTTTGAGTGAGCTGATACCGCTCGCC<br>GCAGCCGAACGACCGAGCGCAGCGAGTCAGTGAGCGAGGAAGCGGAAGAGCAAATA<br>ATAAAAAAGCCGGATTAATAATCTGGCTTTTTATATTCTCTGCCCAATACGCAAACCGC<br>CTCTCCCCGCGCGTTGGCCGATTCATTAATGCAGCTGGCACGACAGGCAAAGACTTCT<br>ACGGTTGGGCAATCGAACGCCACTATTCAGCGCATGACCTGTTTAAGGCGCATAGCTT<br>TGATCATGCCGGTAGAGCACGTTGGGAAGGACATCGTTGGGCACATCGTGAACATGCC<br>AACATGTTTTGGGCGCATGAAGGCATTTGGCGTATCCACTGACT

*Boxes indicate the translational start and stop codons of the protein (sfGFP). Underlines indicate the primer annealing sites. Gray highlight indicates the pEX-A2J2 vector sequence.

Supplementary Table 2. List of plasmids (continued)

No	Name	Sequence (5' to 3')*
4	pEX-A2J2-*oSCRIB*-*SCRIB*-*sfGFP*	GAATTCGAAATTAATACGACTCACTATAGGGGCGGGCGGACGAGCGGGCGGGACGAGCTGAGCAGGA<br>CCAAGCGGGATGCGGAGCCGCCGCCGCCGCCGCTCCCCGCGCTTCCTAAGAGTCTGAGACCCGCCGGG<br>CCGCGCCCCCTGCCCAGGCCCGCAGTCCGAGCGTTCCGAGCGCGGGCCGGGCCCAGCGCCGGCCGCGG<br>CCGCGTCCCCGTCCAGTCTCGGGCGGCCCGCCCCCTGCCTGCGCCCCCTCCCCATGCGCGGCGCCCATG<br>CGGACTGAGCCCCGCCCCCCGGCCCCGAGCCCGCCGAGCGCCGCCGCCGGAGCCCGCGCCGCCCACCC<br>GCACCATGCTCAAGTGCATCCCGCTGTGGCGCTGCAACCGGCACGTGGAGTCGGTGGACAAGCGGCAC<br>TGTTCGCTGCAGGCCGTGCCGGAGGAGATCTACCGCTACAGCCGCAGCCTGGAGGAGCTGCTGCTCGA<br>CGCCAACCAGCTGCGCGAGCTGCCCAAGCCTTTTTCCGGCTGCTGAACTTGCGCAAGCTGGGCCTGAG<br>CGACAACGAGATCCAGCGGTTGCCTCCCGAGGTGGCCAACTTCATGCAGCTGGTGGAGCTGGACGTGT<br>CCCGGAACGATATCCCTGAGGGTTCTGCTGGAAGCGCAGCCGGCTCCGGGGAGTTCTCTAAGGGTGAG<br>GAGCTATTCACAGGCGTGGTACCCATCTTGGTAGAGCTGGACGGGGACGTCAACGGTCACAAATTCTC<br>AGTGCGAGGAGAGGGCGAGGGTGACGCTACTAACGGCAAGTTAACACTGAAGTTTATCTGCACGACAG<br>GCAAGTTGCCCGTTCCTTGGCCAACGCTTGTCACTACCCTGACTTACGGGGTGCAGTGTTTCAGTCGCT<br>ACCCGGACCATATGAAAAGACACGACTTTTTTAAGTCCGCGATGCCCGAAGGCTACGTTCAAGAGCGG<br>ACAATATCCTTTAAAGACGACGGTACCTACAAGACACGTGCCGAAGTCAAGTTCGAGGGAGATACTTT<br>AGTCAACAGGATCGAACTCAAAGGAATTGACTTCAAAGAGGACGGGAATATCCTCGGACATAAACTGG<br>AGTACAACTTTAACTCGCACAACGTTTATATCACTGCCGATAAACAGAAGAACGGAATAAAAGCCAAC<br>TTTAAAATTAGACACAACGTGGAAGACGGAAGCGTGCAGCTCGCAGACCATTATCAACAGAACACCCC<br>CATTGGAGACGGACCAGTGCTGCTGCCAGATAATCATTATCTCTCTACCCAGAGTGTGCTGAGCAAGG<br>ATCCAAACGAAAAGAGGGATCATATGGTGCTGCTTGAGTTCGTGACCGCTGCAGGGATTACCCACGGC<br>ATGGACGAACTTTATAAGGGCTCACACCACCACCATCACCATTAGAGGAGCAGGCACCTCCCCCAGAC<br>TTGGGGTGGGGGCCCTGCCAGCTCCAGCACCACCCTTGCCCCAAGTCTTTTAACCTGGGTGTTAGCATT<br>TTAAAGAGACCCCACAGGAGTTCTGGCCTGTGACTAACTAACTGCCCCACCCCAGCCGAGACCTCGGC<br>GAGACTGTAACTAGTGATGTTTGTACAACCAAAGACTCTATTTTGTGGTTTAAGGAGAATAAAGTTGAC<br>TACATTTTAAAAAAAAAAAAAAAAAAAAAAAAAAAAAAAAAGCTTCTAGCATAACCCCTTGGGGCCT<br>CTAAACGGGTCTTGAGGGGTTTTTTGGTCGACATGCCGTGAGCACAAAAGCGACCATAGTCGGGATTTT<br>ACCCGCTTATCTGCGGAAGGTCATTGGGAACGCATCTTCCACAAATGGGCTGAGCGCAAAAATGCCGA<br>ACGTATCTGGCTGACCTATGCCATTCCGTGGGAACGACACTACTTTTCAATTGGTGCACTCTCAGTACA<br>ATCTGCTCTGATGCCGCATAGTTAAGCCAGCCCCGACACCCGCCAACACCCGCTGACGCGCCCTGACG<br>GGCTTGTCTGCTCCCGGCATCCGCTTACAGACAAGCTGTGACCGTCTCCGGAAAATCAAAGGATCTTCT<br>TGAGATCCTTTTTTTGAGCTGCATGTGTCAGAGGTTTTCACCGTCATCACCGAAACGCGCGAGACGAAA<br>GGGCCTCGTGATACGCCTATTTTTATAGGTTAATGTCATGATAATAATGGTTTCTTAGACGTCAGGTGG<br>CACTTTTCGGGGAAATGTGCGCGGAACCCCTATTTGTTTATTTTTCTAAATACATTCAAATATGTATCCG<br>CTCATGAGACAATAACCCTGATAAATGCTTCAATAATATTGAAAAAGGAAGAGTATGAGTATTCAACA<br>TTTCCGTGTCGCCCTTATTCCCTTTTTTGCGGCATTTTGCCTTCCTGTTTTTGCTCACCCAGAAACGCTGG<br>TGAAAGTAAAAGATGCTGAAGATCAGTTGGGTGCACGAGTGGGTTACATCGAACTGGATCTCAACAGC<br>GGTAAGATCCTTGAGAGTTTTCGCCCCGAAGAACGTTTTCCAATGATGAGCACTTTTAAAGTTCTGCTA<br>TGTGGCGCGGTATTATCCCGTATTGACGCCGGGCAAGAGCAACTCGGTCGCCGCATACACTATTCTCAG<br>AATGACTTGGTTGAGTACTCACCAGTCACAGAAAAGCATCTTACGGATGGCATGACAGTAAGAGAATT<br>ATGCAGTGCTGCCATAACCATGAGTGATAACACTGCGGCCAACTTACTTCTGACAACGATCGGAGGAC<br>CGAAGGAGCTAACCGCTTTTTTGCACAACATGGGGGATCATGTAACTCGCCTTGATCGTTGGGAACCGG<br>AGCTGAATGAAGCCATACCAAACGACGAGCGTGACACCACGATGCCTGTAGCAATGGCAACAACGTTG<br>CGCAAACTATTAACTGGCGAACTACTTACTCTAGCTTCCCGGCAACAATTAATAGACTGGATGGAGGC<br>GGATAAAGTTGCAGGACCACTTCTGCGCTCGGCCCTTCCGGCTGGCTGGTTTATTGCTGATAAATCTGG<br>AGCCGGTGAGCGTGGGAGTCGCGGTATCATTGCAGCACTGGGGCCAGATGGTAAGCCCTCCCGTATCG<br>TAGTTATCTACACGACGGGGAGTCAGGCAACTATGGATGAACGAAATAGACAGATCGCTGAGATAGGT<br>GCCTCACTGATTAAGCATTGGTAACTGTCAGACCAAGTTTACTCATATATACTTTAGATTGATTTAAAA<br>CTTCATTTTTAATTTAAAAGGATCTAGGTGAAGATCCTTTTTGATAATCTCATGACCAAAATCCCTTAAC<br>GTGAGTTTTCGTTCCACTGAGCGTCAGACCCCGTAGAAAAGATCAAAGGATCTTCTTGAGATCCTTTTT<br>TTCTGCGCGTAATCTGCTGCTTGCAAACAAAAAAACCACCGCTACCAGCGGTGGTTTGTTTGCCGGATC<br>AAGAGCTACCAACTCTTTTTCCGAAGGTAACTGGCTTCAGCAGAGCGCAGATACCAAATACTGTTCTTC<br>TAGTGTAGCCGTAGTTAGGCCACCACTTCAAGAACTCTGTAGCACCGCCTACATACCTCGCTCTGCTAA<br>TCCTGTTACCAGTGGCTGCTGCCAGTGGCGATAAGTCGTGTCTTACCGGGTTGGACTCAAGACGATAGT<br>TACCGGATAAGGCGCAGCGGTCGGGCTGAACGGGGGGTTCGTGCACACAGCCCAGCTTGGAGCGAAC<br>GACCTACACCGAACTGAGATACCTACAGCGTGAGCTATGAGAAAGCGCCACGCTTCCCGAAGGGAGAA<br>AGGCGGACAGGTATCCGGTAAGCGGCAGGGTCGGAACAGGAGAGCGCACGAGGGAGCTTCCAGGGGG<br>AAACGCCTGGTATCTTTATAGTCCTGTCGGGTTTCGCCACCTCTGACTTGAGCGTCGATTTTTGTGATGC<br>TCGTCAGGGGGGCGGAGCCTATGGAAAAACGCCAGCAACGCGGCCTTTTTACGGTTCCTGGCCTTTTGC<br>TGGCCTTTTGCTCACATGTTCTTTCCTGCGTTATCCCCTGATTCTGTGGATAACCGTATTACCGCCTTTGA<br>GTGAGCTGATACCGCTCGCCGCAGCCGAACGACCGAGCGCAGCGAGTCAGTGAGCGAGGAAGCGGAA<br>GAGCAAATAATAAAAAAGCCGGATTAATAATCTGGCTTTTTATATTCTCTGCCCAATACGCAAACCGCC<br>TCTCCCCGCGCGTTGGCCGATTCATTAATGCAGCTGGCACGACAGGCAAAGACTTCTACGGTTGGGCAA<br>TCGAACGCCACTATTCAGCGCATGACCTGTTTAAGGCGCATAGCTTTGATCATGCCGGTAGAGCACGTT<br>GGGAAGGACATCGTTGGGCACATCGTGAACATGCCAACATGTTTTGGGCGCATGAAGGCATTTGGCGT<br>ATCCACTGACT

*Boxes indicate the translational start and stop codons of the proteins (oSCRIB and SCRIB-sfGFP). Green, pink, and yellow highlights correspond to *SCRIB* (C-terminally truncated), flexible linker, and *sfGFP* sequences, respectively. Underlines indicate the primer annealing sites. Gray highlight indicates the pEX-A2J2 vector sequence.

Supplementary Table 3. List of PCR primers

No	Primer name	Primer sequence (5' to 3')
1	Fw1-S	GAAATAATTTTGTTTAACTTTAAGAAGGAGATATACCAATGGGACAT CATCATCATCATCACGATTACG
2	Fw1-G	GAAATAATTTTGTTTAACTTTAAGAAGGAGATATACCAATGTCTAAG GGAGAAGAACTGTTCACC
3	Fw1-SG1	CATTTTACATTCTACAACTACATAACTAACTAGTAACAATGCGGACT GAGCCCCGCCCCCCGGCCCCG
4	Fw1-SG2	CATTTTACATTCTACAACTACATAACTAACTAGTAACAAGGCGGACT GAGCCCCGCCCCCCGGCCCCG
5	Fw1-SG3	CATTTTACATTCTACAACTACATAACTAACTAGTAACACCCCGGACT GAGCCCCGCCCCCCGGCCCCG
6	Fw1-SG4	CATTTTACATTCTACAACTACATAACTAACTAGTAACAATGCTCAAG TGCATCCCGCTGTGGCGCTGC
7	Rv1	CAGTGAGGCACCTATCTCAGCG
8	Fw2-E	CGACTCACTATAGGGAGACCACAACGGTTTCCCTCTAGAAATAATTT TGTTTAACTTTAAGAAGGAGATA
9	Fw3-E	GAAATTAATACGACTCACT
10	Fw2-W	GGTGACACTATAGAAGTATTTTTACAACAATTACCAACAACAACAAC AAACAACAACAACATTACATTTTACATTCTACAACTACATAACTAAC
11	Fw3-W	GCGTAGCATTTAGGTGACACT
12	Rv2	CTGTCTATTTCGTTCATCCATAGTTGCCTG

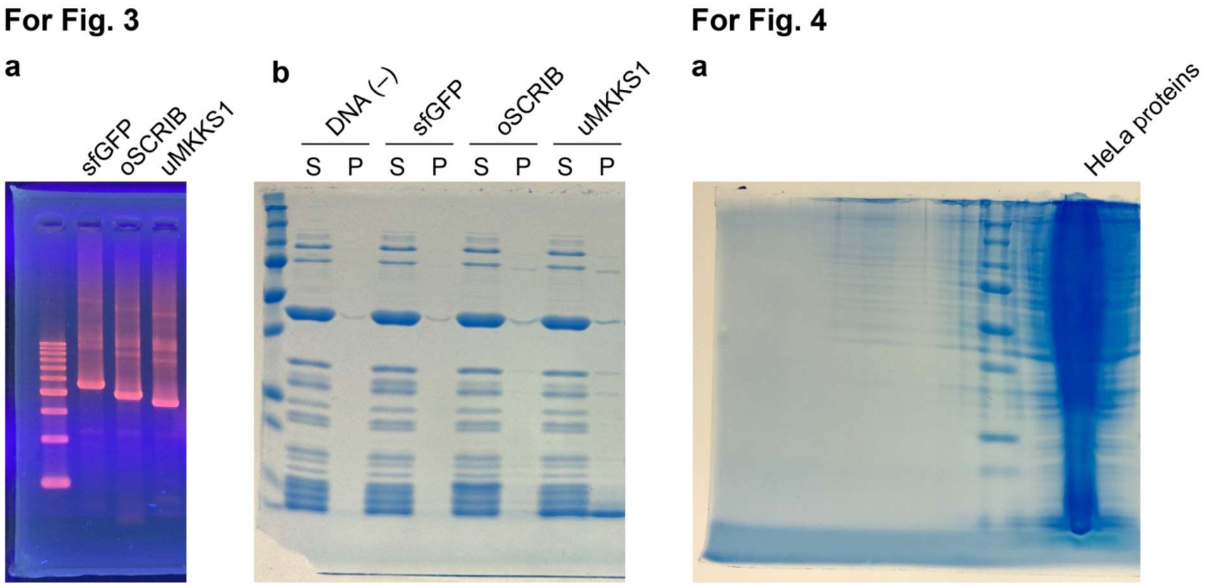

Supplementary Figure 1. Uncropped and unedited gel images.
