# Supplementary material for: Discovery of a small protein-encoding cis-regulatory overlapping gene of the tumor suppressor gene Scribble in humans
Source: Commun Biol. 2021 Sep 17;4:1098. doi: 10.1038/s42003-021-02619-8 (PMC8448870; doi:10.1038/s42003-021-02619-8)
Supplement: Supplementary file 2 — Description of Additional Supplementary Files [file 42003_2021_2619_MOESM2_ESM.pdf]

Description of Additional Supplementary Files

File name: Supplementary Data 1

Description: Source data underlying graphs and charts in Figures 5a, 5b, and 6b.
