# Supplementary material for: Discovery of a small protein-encoding cis-regulatory overlapping gene of the tumor suppressor gene Scribble in humans
Source: Commun Biol. 2021 Sep 17;4:1098. doi: 10.1038/s42003-021-02619-8 (PMC8448870; doi:10.1038/s42003-021-02619-8)
Supplement: Supplementary file 4 — Reporting Summary [file 42003_2021_2619_MOESM4_ESM.pdf]

nature research

Corresponding author(s): Yuhta Nomura and Naoshi Dohmae

Last updated by author(s): Aug 11, 2021

Reporting Summary

Nature Research wishes to improve the reproducibility of the work that we publish. This form provides structure for consistency and transparency in reporting. For further information on Nature Research policies, see our Editorial Policies and the Editorial Policy Checklist.

Statistics

For all statistical analyses, confirm that the following items are present in the figure legend, table legend, main text, or Methods section.

n/a	Confirmed	
☐	☒	The exact sample size (n) for each experimental group/condition, given as a discrete number and unit of measurement
☐	☒	A statement on whether measurements were taken from distinct samples or whether the same sample was measured repeatedly
☒	☐	The statistical test(s) used AND whether they are one- or two-sided<br>*Only common tests should be described solely by name; describe more complex techniques in the Methods section.*
☒	☐	A description of all covariates tested
☒	☐	A description of any assumptions or corrections, such as tests of normality and adjustment for multiple comparisons
☐	☒	A full description of the statistical parameters including central tendency (e.g. means) or other basic estimates (e.g. regression coefficient) AND variation (e.g. standard deviation) or associated estimates of uncertainty (e.g. confidence intervals)
☒	☐	For null hypothesis testing, the test statistic (e.g. F, t, r) with confidence intervals, effect sizes, degrees of freedom and P value noted<br>*Give P values as exact values whenever suitable.*
☒	☐	For Bayesian analysis, information on the choice of priors and Markov chain Monte Carlo settings
☒	☐	For hierarchical and complex designs, identification of the appropriate level for tests and full reporting of outcomes
☒	☐	Estimates of effect sizes (e.g. Cohen's d, Pearson's r), indicating how they were calculated

Our web collection on statistics for biologists contains articles on many of the points above.

Software and code

Policy information about availability of computer code

Data collection	NCBI SRA Toolkit 2.9.6-1, Thermo Xcalibur, and Wallac 1420 Manager 3.00.
Data analysis	Trim Galore! 0.6.2, Trinity 2.8.5, TransDecoder.LongOrfs 5.5.0, SeqKit 0.12.0, BLAST+ 2.9.0, Thermo Proteome Discoverer 2.2.0.388, Mascot Server 2.7.0, PepQuery 1.6.0, Thermo Xcalibur Qual Browser 3.1.66.10, Integrative Proteomics Data Viewer (PDV) 1.6.0, and Integrative Genomics Viewer (IGV) 2.8.0.

For manuscripts utilizing custom algorithms or software that are central to the research but not yet described in published literature, software must be made available to editors and reviewers. We strongly encourage code deposition in a community repository (e.g. GitHub). See the Nature Research guidelines for submitting code & software for further information.

Data

Policy information about availability of data

All manuscripts must include a data availability statement. This statement should provide the following information, where applicable:

- Accession codes, unique identifiers, or web links for publicly available datasets
- A list of figures that have associated raw data
- A description of any restrictions on data availability

All data supporting the findings of this study are available within the paper and its supplementary information file. Uncropped and unedited gel images were included in Supplementary Figure 1. Source data for graphs were included in Supplementary Data 1. The protein sequence data reported in this paper will appear in the UniProt Knowledgebase under the accession number C0HLS1 for oSCRIB in humans (Homo sapiens). The raw mass spectrometric data and Mascot-related files have been deposited to the ProteomeXchange Consortium via the PRIDE partner repository with the dataset identifier PXD027841 and 10.6019/PXD027841.

Field-specific reporting

Please select the one below that is the best fit for your research. If you are not sure, read the appropriate sections before making your selection.

☒ Life sciences ☐ Behavioural & social sciences ☐ Ecological, evolutionary & environmental sciences

For a reference copy of the document with all sections, see nature.com/documents/nr-reporting-summary-flat.pdf

Life sciences study design

All studies must disclose on these points even when the disclosure is negative.

Sample size	No statistical methods were used to predetermine sample sizes. LC-MS/MS analyses were performed at least three times with the same preparation of each protein/fraction and similar results were obtained. Fluorescence analyses were performed three times with different preparations of each protein.
Data exclusions	No data were excluded from the analyses.
Replication	LC-MS/MS analyses were performed at least three times with the same preparation of each protein/fraction and similar results were obtained. Fluorescence analyses were performed three times with different preparations of each protein.
Randomization	No randomized trials were performed in this study. These trials are not conventionally used in such studies.
Blinding	No blinded experiments were performed in this study. These experiments are not conventionally used in such studies.

Reporting for specific materials, systems and methods

We require information from authors about some types of materials, experimental systems and methods used in many studies. Here, indicate whether each material, system or method listed is relevant to your study. If you are not sure if a list item applies to your research, read the appropriate section before selecting a response.

Materials & experimental systems

n/a	Involved in the study	
☒	☐	Antibodies
☐	☒	Eukaryotic cell lines
☒	☐	Palaeontology and archaeology
☒	☐	Animals and other organisms
☒	☐	Human research participants
☒	☐	Clinical data
☒	☐	Dual use research of concern

Methods

n/a	Involved in the study	
☒	☐	ChIP-seq
☒	☐	Flow cytometry
☒	☐	MRI-based neuroimaging

Eukaryotic cell lines

Policy information about cell lines

Cell line source(s)	HeLa cells were provided by Dr. Muroi, Chemical Biology Group, RIKEN Center for Sustainable Resource Science, Japan (Muroi et al., 2010; Muroi and Osada, 2019).
Authentication	The cell line was authenticated by short tandem repeat (STR) analysis. In addition, the identity of the cell line was frequently checked by its morphological feature. The cells did not show any signs of contamination.
Mycoplasma contamination	Mycoplasma contamination was not tested in this study.
Commonly misidentified lines (See ICLAC register)	No commonly misidentified cell lines were used in this study.
